# Traditional Chinese Medicine Borneol‐Based Polymeric Micelles Intracerebral Drug Delivery System for Precisely Pathogenesis‐Adaptive Treatment of Ischemic Stroke

**DOI:** 10.1002/advs.202410889

**Published:** 2025-01-13

**Authors:** Yanan Wang, Xutao Ma, Xinyuan Wang, Liru Liu, Xue Zhang, Qiuyue Wang, Yingfei Zhu, Huanhua Xu, Liangmin Yu, Zhiyu He

**Affiliations:** ^1^ Frontiers Science Center for Deep Ocean Multispheres and Earth Systems Key Laboratory of Marine Chemistry Theory and Technology Ministry of Education/Sanya Oceanographic Institution Ocean University of China Qingdao/Sanya 266003/572024 China; ^2^ Sanya Oceanographic Laboratory Sanya 572024 China; ^3^ College of Chemistry and Chemical Engineering Ocean University of China Qingdao 266003 China; ^4^ Key Laboratory of Modern Preparation of TCM Ministry of Education Jiangxi University of Chinese Medicine Nanchang 330004 China; ^5^ State Key Laboratory for the Modernization of Classical and Famous Prescriptions of Chinese Medicine Jiangxi University of Chinese Medicine Nanchang 330004 China

**Keywords:** BAPTA‐AM, blood‐brain barrier, borneol, ischemic stroke, ROS‐responsive

## Abstract

The scarcity of effective neuroprotective agents and the presence of blood‐brain barrier (BBB)‐mediated extremely inefficient intracerebral drug delivery are predominant obstacles to the treatment of cerebral ischemic stroke (CIS). Herein, ROS‐responsive borneol‐based amphiphilic polymeric NPs are constructed by using traditional Chinese medicine borneol as functional blocks that served as surface brain‐targeting ligand, inner hydrophobic core for efficient drug loading of membrane‐permeable calcium chelator BAPTA‐AM, and neuroprotective structural component. In MCAO mice, the nanoformulation (polymer: 3.2 mg·kg^−1^, BAPTA‐AM: 400 µg·kg^−1^) reversibly opened the BBB and achieved high brain biodistribution up to 12.7%ID/g of the total administered dose after 3 h post single injection, effectively restoring intracellular Ca^2+^ and redox homeostasis, improving cerebral histopathology, and inhibiting mitochondrial PI3K/Akt/Bcl‐2/Bax/Cyto‐C/Caspase‐3,9 apoptosis pathway for rescuing dying neurons (reduced apoptosis cell from 59.5% to 7.9%). It also remodeled the inflammatory microenvironment in cerebral ischemic penumbra by inhibiting astrocyte over‐activation, reprogramming microglia polarization toward an anti‐inflammatory phenotype, and blocking NF‐κB/TNF‐*α*/IL‐6 signaling pathways. These interventions eventually reduced the cerebral infarction area by 96.3%, significantly improved neurological function, and restored blood flow reperfusion from 66.2% to ≈100%, all while facilitating BBB repair and avoiding brain edema. This provides a potentially effective multiple‐stage sequential treatment strategy for clinical CIS.

## Graphical abstract

Traditional Chinese medicine borneol as brain‐targeted and therapeutic functional blocks of ROS‐responsive polymeric NPs. The responsively released calcium chelator BAPTA‐AM effectively inhibit Ca^2+^ overload in injured neurons. Dissociated borneol synergistically restore redox homeostasis, inhibit inflammatory cascade and promote BBB repair. This formulation promotes multiple‐stage sequential treatment strategy in ischemic stroke.

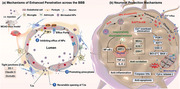

## 1 Introduction

Stroke is a prevalent cerebrovascular disease that is associated with high mortality and severe disability (>65%).^[^ ^1^ ^]^ Cerebral ischemia stroke (CIS) accounts for more than 80% of all strokes, resulting in an irreversible injury to the infarct core and a salvageable surrounding penumbra in the ischemic hemisphere. Furthermore, recanalization of obstructed blood vessels invariably leads to deleterious secondary cerebral ischemia/reperfusion (I/R) injury, thereby expanding the infarct area while exacerbating neuronal damage and functional impairments. Unfortunately, there are less breakthroughs in the development of innovative drugs and clinical therapies with effective therapeutic efficiency with multi‐stage sequential therapy in cerebral I/R injury.^[^ ^2^ ^]^

The pathophysiological process of CIS primarily exhibits the following characteristics: 1) nerve excitotoxicity‐mediated upstream intracellular calcium overload, followed by oxidative stress and energy metabolism disorders, which are mutually feedback‐regulated cell damage cascades that could exacerbate neuronal damage;^[^ ^3^ ^]^ 2) neuroinflammation cascade, which is mediated by significant amounts of inflammatory mediators that are released from overactivated microglia and astrocytes;^[^ ^4^ ^]^ and 3) endothelial injury and microcirculation disorder in the late perfusion period harm blood‐brain barrier (BBB) function and produce cerebral edema.^[^ ^5^ ^]^ Conversely, the neuroprotective drugs that are currently being studied or in clinical trials have limited efficacy as a result of their low solubility, short half‐lives, and the fact that they only target a single stage of pathology.^[^ ^6^ ^]^ Additionally, they may not be able to simultaneously repair the injured BBB and inhibit brain edema during the late stages of reperfusion.^[^ ^7^ ^]^ Most importantly, the existence of BBB results in extremely low drug brain delivery efficiency (<1%), making it difficult to achieve effective therapeutic concentrations. Nano‐engineered drug delivery systems (DDSs) that have been recently developed are designed to enhance drug transport across the BBB through mechanisms like adsorption‐mediated endocytosis, receptor‐mediated endocytosis, cell‐mediated transport, and so on.^[^ ^8^ ^]^ Nevertheless, they are still confronting challenges, including restricted brain targeting, inefficient BBB penetration, high cytotoxicity, potential disruption of BBB integrity, poor storage stability, and the challenges of large‐scale production.^[^ ^9^ ^]^

Borneol (BO), a lipid‐soluble bicyclic monoterpene, has been clinically used in traditional Chinese medicine for over 1500 years to restore consciousness, provide refreshment, and have antipyretic and analgesic effects, particularly in the treatment of encephalopathy.^[^ ^10^ ^]^ Borneol has been reported to reversibly open the BBB, enhance microcirculatory perfusion, and have anti‐coagulation and anti‐inflammatory properties, etc., serving multiple functions in injured brain tissue.^[^ ^11^ ^]^ However, the detailed mechanism of borneol's action on the BBB opening, its specific cell selectivity, the molecular mechanism of intervention processes involved in the overall progression of cerebral injury, and the optimal therapeutic dosage have not yet been thoroughly identified. Borneol is typically administered orally in oil or capsules at high dosages (>200 mg·kg^−1^) for a long‐term, repeated period (3–8 days) as a prophylactic treatment for CIS management in preclinical experiments.^[^ ^12^ ^]^ However, during a clinically acute sudden stroke event, it is virtually impossible to rapidly and effectively rescue damaged cerebral ischemic penumbra using current dosing regimens.^[^ ^13^ ^]^ Excessive borneol administration can result in acute central nervous system overexcitability, tachycardia, arrhythmia, an allergic reaction, severe gastric mucosal disruption, liver and kidney abnormalities, and even reproductive toxicity. Besides, borneol is ineffective in blocking the simultaneous and continuous activation of multiple downstream cell death pathways in neurons, which is initiated by upstream intracellular calcium overload.

Excitingly, a hydrophobic cell membrane‐permeable calcium chelator, 1,2‐bis‐(2‐aminophenoxy) ethane‐*N*, *N*, *N*′, *N*′‐tetraacetic acid acetoxymethyl ester (BAPTA‐AM, i.e., BA‐AM), could effectively and selectively chelate intracellular excess Ca^2+^ in the form of BAPTA post‐hydrolysis by intracellular esterase without undergoing protonation in the blood, demonstrating high stability and safety during circulation.^[^ ^14^ ^]^ However, its therapeutic applicability remains restricted due to its low water solubility, short half‐life, and lack of organ‐targeting specificity.

The study aims to covalently conjugate the borneol molecule with FDA‐approved polyethylene glycol (PEG) as hydrophilic blocks (methacrylate‐PEG‐Borneol, MPB) and with ROS‐cleavable thioketal (TK) bond linkers as hydrophobic blocks (methacrylate‐TK‐Borneol, MTB), respectively. These monomers would then be polymerized via RAFT polymerization at a tunable hydrophilic/hydrophobic monomer ratio, yielding an amphiphilic borneol‐based co‐polymer p(PB)_10_/(TB)_30_ featuring a straightforward and customizable chemical structure with a single borneol active constituent, where borneol serves as both a brain‐targeting ligand, a hydrophobic functional structure, and a neuroprotective component. The amphiphilic copolymer would self‐assemble into polymer prodrug NPs with controlled particle size, narrow size distribution, and high loading of the model drug BAPTA‐AM by accurately designing and controlling the self‐assembly process in a well‐defined and ordered manner using the flash nanocomplexation (FNC) approach.

**Scheme 1:**
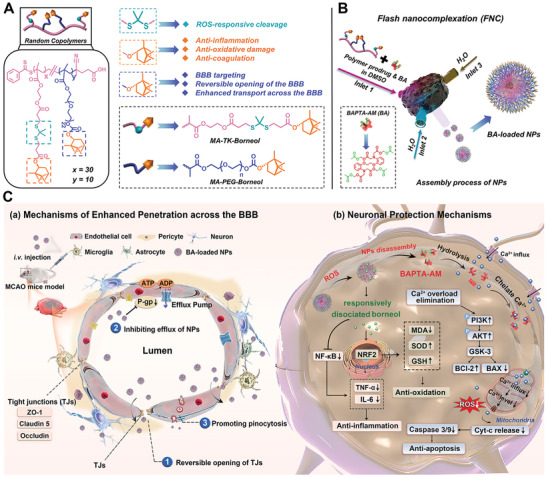
A schematic representation depicting A) the chemical structure of p(PB)_10_/(TB)_30_ polymer and the functions of different structures; B) the FNC setup for the preparation of BA‐loaded NPs. The inlet 1 was designated for feeding the polymer and BAPTA‐AM dissolved in DMSO (organic phase), while inlets 2 and 3 were utilized for feeding water (aqueous phase), and C) their BBB‐penetrating and therapeutic mechanisms in a middle cerebral artery occlusion (MCAO) mouse model.

The polymer prodrug NPs are designed to seamlessly integrate multiple functionalities, such as prolonged blood circulation, rapid and efficient crossing of the BBB to reach the cerebral ischemic penumbra without compromising BBB integrity, and the ability to dynamically dissociate borneol while simultaneously releasing the model drug payload (BAPTA‐AM) in intracellular ROS‐rich pathological microenvironments. Following hydrolysis in the neuron, the BAPTA would rapidly eliminate the intracellular calcium overload upstream of the cell injury cascade, interrupting the calcium overload‐mediated feedback downstream continuous activation regulatory loops of severe oxidative stress and energy metabolism disorder, thereby preventing the downstream cell apoptosis signaling pathway. The dissociated active borneol fully intervenes in the I/R injury cascade at multiple levels, including improving microcirculation, inhibiting the inflammatory cascade, scavenging the excess ROS, and restoring the structure and function of the compromised BBB during the late reperfusion period. This formulation integrates “reversible opening of the BBB”, “precisely controlled drug release”, “ion homeostasis regulation”, “restoring consciousness” and “repairing the BBB” to achieve multi‐stage sequential therapy of cerebral I/R injury, which provides collaborative, systemic, and comprehensive interventions in CIS management (**Scheme** **1**).

## 2 Results

### 2.1 Preparation and Characterization of Polymer Prodrug NPs

#### 2.1.1 Synthesis of Borneol‐Based Polymers

Figures S1 and S2 (Supporting Information) provide detailed illustrations of the synthetic procedures. ROS‐cleavable hydrophobic methacrylate‐TK‐Borneol (MTB) monomers were synthesized by conjugating borneol to 2‐hydroxyethyl methacrylate via a TK linker (Figures S3–S5, Supporting Information). The two small peaks observed at 4.5‐5.0 ppm were ascribed to the proton peak of CH after the hydroxyl group of borneol, which is coupled with the carboxyl group of TK. Additionally, the three distinct peaks at 0.9 ppm in the spectrum correspond to the methyl proton peaks of borneol, suggesting successful coupling of borneol (Figure S5, Supporting Information). Furthermore, borneol was conjugated to methacrylate‐PEG (MP), synthesized by reacting PEG (Mn: 1.0 kDa) with methacrylic anhydride, yielding hydrophilic MP‐Borneol (MPB) monomers (Figures S6 and S7, Supporting Information). The peaks at 5.6 ppm and 6.1 ppm correspond to the proton signals of olefins, indicating the coupling of hydroxyethyl methacrylate. The proton peak at 1.9 ppm, associated with the methyl group, the proton peak at 3.65 ppm from the repeating unit in the PEG chain, and the multiple peaks at 4.28 ppm corroborated the formation of the hydrophilic MPB monomer. Thereafter, the amphiphilic p(PB)_10_/(TB)_30_ was synthesized via RAFT polymerization of MTB and MPB monomers. The ^1^H NMR spectrum of the random copolymer includes all the characteristic peaks of borneol (h) and PEG (i), corresponding to the proton peak at 0.75‐1 ppm and the single peak at 3.65 ppm, respectively, thereby confirming the synthesis of borneol‐based polymers (Figure S8, Supporting Information). The gel permeation chromatography (GPC) measurements confirmed successful polymerization, revealing that the co‐polymer prodrug had a well‐defined monomodal and narrow molecular weight distribution (Mw/Mn = 1.46), with a number‐average molecular weight of 24 kDa (Figure S9, Supporting Information). The control polymer, p(P)_10_/(TB)_30_, was prepared via direct RAFT polymerization of MTB and MP in accordance with the same procedures.

#### 2.1.2 Parameter Optimization of NPs

##### Optimization of FNC Parameters to Generate Uniform NPs

**Figure 1:**
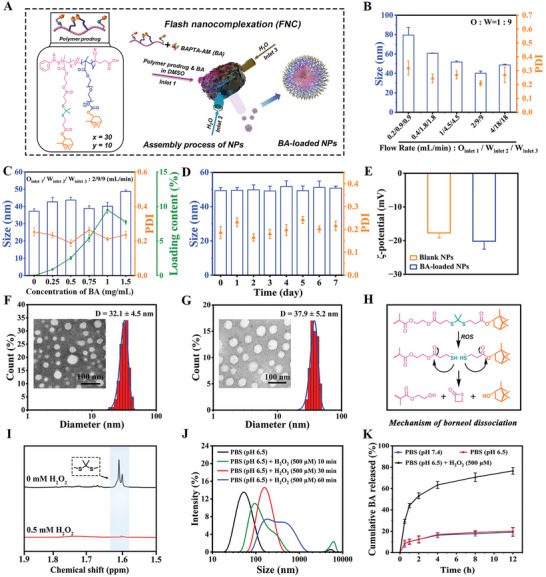
Preparation and characterization of BA‐loaded NPs. A) The polymer prodrug's chemical structure and the FNC setup for preparation of NPs. The inlet 1 was designated for feeding the polymer and BAPTA‐AM dissolved in DMSO (organic phase), while inlets 2 and 3 were utilized for feeding water (aqueous phase). B) The effect of the total volumetric flow rate on the size distribution of NPs. The organic phase's volumetric flow rate ratio to the aqueous phase was set at 1:9. C) The effect of the mass ratio of the polymer with BA on average size and LC. The volumetric flow rate in inlet 1, 2, and 3 was set at 2, 9, and 9 mL·min^−1^, respectively. D) Stability of BA‐loaded NPs incubated in stimulated physiological PBS medium (10 mM, pH 7.4) at 25 °C. E) The ζ‐potential of blank NPs and BA‐loaded NPs. Representative transmission electron microscope (TEM) image and quantitative particle diameter distribution results of F) blank NPs and G) BA‐loaded NPs. Scale bar: 100 nm. H) A schematic diagram of the mechanism of responsive dissociation of borneol from hydrophobic block under ROS stimulation. I) Conversion of polymer to polymeric fragments under the action of 0.5 mM H_2_O_2_ analyzed by ^1^H NMR spectra. The inset only showed partial chemical shifts between 1.5 and 1.9 ppm. J) Size distributions of BA‐loaded NPs after exposure to PBS medium (pH 6.5) with or without 500 µM H_2_O_2_. K) Drug release profiles of formulation in PBS (pH 6.5 or 7.4) and PBS (pH 6.5) containing 500 µM H_2_O_2_. n = 3. Data are shown as mean ± SEM.

To kinetically control the particle assembly process and continuously produce NPs with controlled size, high encapsulation efficiency, as well as good reproducibility, the p(PB)_10_/(TB)_30_ NPs were prepared using a FNC method for BAPTA‐AM (BA) loading. When the ratio of organic (O) to water phase (W) fixed at 1:9 and BA concentration was fixed at 1 mg·mL^−1^, size of particles decreased dramatically from 80.2 to 44.9 nm with total flow rate increasing from 2.0 mL·min^−1^ (O_inlet1_: 0.2 mL·min^−1^, W_inlet2_: 0.9 mL·min^−1^, W_inlet3_: 0.9 mL·min^−1^, respectively) to 20 mL·min^−1^ (O_inlet1_: 2.0 mL·min^−1^, W_inlet2_: 9.0 mL·min^−1^, W_inlet3_: 9.0 mL·min^−1^, respectively), while the ptransmission electron microscopeolydispersity index (PDI) dropped from 0.318 to 0.209 (**Figure** **1**B). The as‐prepared NPs exhibited a slightly larger particle size when the total flow rate was further increased from 20 to 40 mL·min^−1^ (O_inlet1_: 4 mL·min^−1^, W_inlet2_: 18 mL·min^−1^, W_inlet3_: 18 mL·min^−1^, respectively); however, this did not significantly improve particle uniformity (PDI rebounded to 0.269). Subsequently, the O/W ratio of 1:9 and total flow rate of 20 mL·min^−1^ was chosen as the preparation parameters.

##### Optimization of the EE of BA

The loading content (LC%) increased significantly as the feeding drug concentrations in FNC increased (from 0.25 to 1 mg·mL^−1^), reaching a plateau at 9.5% at 1 mg·mL^−1^. However, this did not have a significant impact on the particle size distribution (Figure 1C). Additional increases in the feeding BA concentrations (from 1 to 1.5 mg·mL^−1^) result in a rebound trend, which causes a notable decline in encapsulation efficiency (EE%dynamic light scattering) from 86.5% to 44.8% and a rebound trend, evidenced by a reduction in LC% to 7.7%, indicating that the encapsulation of BA has surpassed its saturation point within the polymer matrix. This phenomenon can be ascribed to the elevated drug concentration in the organic phase, which results in an increased diffusion rate of the drug into water, hence allowing a larger portion of the drug to exit the organic phase without being encapsulated prior to NPs formation.^[^ ^15^ ^]^ As anticipated, when the feeding BA concentration reached 1.5 mg·mL^−1^, the as‐prepared nanosuspension was observed to precipitate within the following hour.

##### Batch‐to‐Batch Preparation Reproducibility

The NPs showed nearly similar average size, PDI, and EE by collecting samples at different output flow volumes after stable flow in the FNC and uniform mixing process, as well as different batches upon preparation on various days, indicating a highly consistent and scalable properties of the particle fabrication process (Tables S1 and S2, Supporting Information).

#### 2.1.3 NPs Characterization

The dynamic light scattering (DLS) results revealed that the optimized blank NPs and BA‐loaded NPs exhibited small sizes (37.2 nm and 42.3 nm, respectively), uniform size distribution (PDI: 0.228 and 0.212, respectively), and negative surface charge (ζ‐potential: ‐17.7 and ‐20.2 mV, respectively) (Figure 1E; Figure S10, Supporting Information). Stability studies revealed no significant changes in the size distribution of blank NPs and BA‐loaded NPs after incubation for more than 7 days in both water and a PBS medium (10 mM, pH 7.4), indicating good storage stability in the form of NPs suspension and colloidal stability under physiological conditions (Figure 1D; Figures S11 and S12, Supporting Information). Additionally, TEM images showed that both blank or BA‐loaded NPs had a uniform spherical structure, with average sizes of 32.1 ± 4.5 nm and 37.9 ± 5.6 nm, respectively, slightly smaller than hydrodynamic diameter of the NPs suspension analyzed by DLS measurements (Figure 1F,G). In order to verify its targeting in the following experiments, the Cy5 fluorescent dye was employed to label the p(P)_10_/(TB)_30_, p(PB)_10_/(TB)_30_, and PEG‐PLGA polymers. These polymers were also prepared using the FNC method, yielding NPs with a small size (40–45 nm) and a narrow size distribution (PDI: ≈0.210) that were comparable to BA‐loaded NPs (Figure S13, Supporting Information).

#### 2.1.4 ROS‐Responsive NPs Disassembly and Drug Release Profiles

The methyl peak of TK in the polymer at 1.5–1.9 ppm in the ^1^H NMR spectra had almost completely disappeared following 0.5 mM H_2_O_2_ incubation, indicating the oxidative cleavage of the TK linker (Figure 1H,I). After 10 min of incubating BA‐loaded NPs with simulated pathological PBS medium (pH 6.5) containing 0.5 mM H_2_O_2_, particle size significantly increased from 42.3 to 137.0 nm (Figure 1J). Over time (60 min), the NPs displayed multiple peaks in size distribution, shifting from a distinct unimodal to a wide bimodal distribution, while the “Tyndall Effect” light path of the NPs suspension gradually blurred, revealing the visible movement of solid particles in the light path (Figure S14, Supporting Information). Additionally, following 60 min of H_2_O_2_ exposure, TEM images of NPs revealed visible irregular particle morphology, enlarged diameters, and aggregation of incompact particles (Figure S15, Supporting Information). These findings can be attributed to ROS‐responsive TK bond cleavage and subsequent hydrophobic borneol dissociation, which led to the destruction of the hydrophobic region. This resulted in a looser, unstable, and even disintegrated structure of particles in a short period of time, while partially unstable particles and particle fragments tended to aggregate rapidly. Figure 1K depicts that there was little drug leakage in PBS medium, with less than 10% and 20% of the cumulative drug release amount within 1 and 12 h, respectively. Notably, in a simulated pathological microenvironment (PBS medium with 0.5 mM H_2_O_2_, pH 6.5), the cumulative drug release amount reached ≈44.1% within 1 h and ≈76.6% over a 12‐h period, implying significantly accelerated drug release in ROS‐rich microenvironments.

### 2.2 Effective Drug Transport Across the BBB In Vitro

#### 2.2.1 Reversible Opening of the Tight Junction

**Figure 2:**
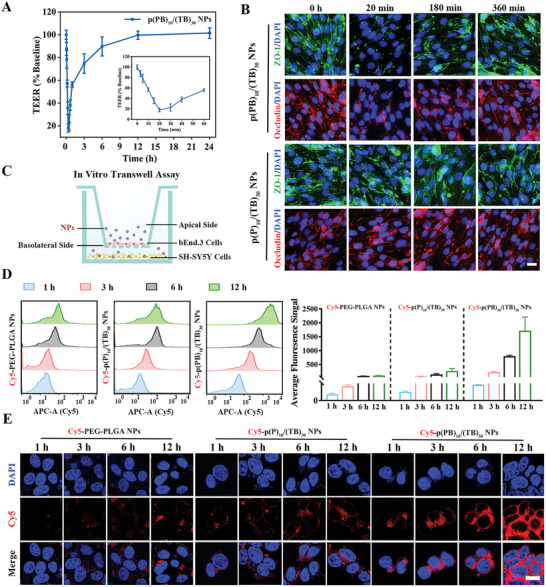
p(PB)_10_/(TB)_30_ NPs reversibly open tight junctions (TJs) and promote transport across the BBB. A) Changes in the transmembrane resistance (TEER) value of BBB‐mimic bEnd.3 monolayers at different time points after incubation with p(PB)_10_/(TB)_30_ NPs. *n* = 3. B) Representative fluorescent image of the variations in ZO‐1 proteins and Occludin proteins over time in BBB‐mimic bEnd.3 monolayers, following incubation with p(PB)_10_/(TB)_30_ NPs or p(P)_10_/(TB)_30_ NPs. Scale bar: 20 µm. n = 6. C) Scheme of an in vitro transwell study on co‐culture of bEnd.3 cells (up) and SH‐SY5Y cells (down). NPs were added to the upper BBB‐mimic bEnd.3 monolayer. D) The cell uptake results of Cy5‐labaled NPs in SH‐SY5Y cells after crossing the BBB‐mimic bEnd.3 monolayers, assessed using flow cytometry. n = 3. E) The cell uptake results of Cy5‐labaled NPs in SH‐SY5Y cells after crossing the BBB‐mimic bEnd.3 monolayers, assessed using confocal microscopy imaging. Scale bar: 10 µm. n = 6. Data are shown as mean ± SEM.

The cytotoxicity test demonstrated that the as‐prepared NPs caused negligible impact on cell viability at polymer concentrations ranging from 6.25 to 800 µg·mL^−1^, indicating acceptable biosafety (Figure S16, Supporting Information). We assessed the transmembrane epithelial electric resistance (TEER) of the BBB‐mimic bEnd.3 monolayer after NPs treatment, as this is the most sensitive and traditional indicator of BBB barrier permeability. A decrease in this value indicates that ions are passing through the paracellular pathway during TJ opening.^[^ ^16^ ^]^ Exposure to p(PB)_10_/(TB)_30_ NPs resulted in a rapid decline in TEER levels within a short period (≈20 min), reaching levels that were 17.9% of the original baseline values (**Figure** **2**A). The TEER value then gradually recovered, returning to 74.7% after only 3 h and ≈100% of the baseline after 12 h. It is hypothesized that borneol on the particle surface temporarily widens the TJs’ gap, allowing for rapid and efficient penetration across the BBB‐mimic bEnd.3 cell monolayer by our prepared NPs with a smaller size (≈38 nm) and reaching the bottom of the Transwell chamber. As NPs penetrating the BBB increasing, the limited number of remaining NPs may not be sufficient to maintain the TJs’ connection open continuously, potentially restoring the TEER value and the barrier functionality of the BBB.

After 20 min of incubation with p(P)_10_/(TB)_30_ NPs, immunofluorescent straining exhibited no significant changes in the arrangements of ZO‐1 and Occludin proteins in intercellular TJs’ complexes (Figure 2B; Figures S17 and S18, Supporting Information). Comparatively, following co‐incubation with p(PB)_10_/(TB)_30_ NPs, both the arrangements of ZO‐1 and Occludin proteins appeared obscure and discontinuous, implying that the presence of borneol targets facilitates translocation of TJs proteins from cell membrane to cytoplasm, ultimately leading to an increase in barrier permeability (Figure 2B; Figures S18 and S20, Supporting Information). As expected, at 3 and 6 h, the arrangement morphology of ZO‐1 and Occludin reverted to its original continuous ring feature surrounding cell monolayer boundaries, implying a recovery of robust and highly restricted endothelial barrier function.

#### 2.2.2 Effective Penetration Across the BBB

We constructed an in vitro BBB Transwell model and added the NPs into the upper BBB‐mimic bEnd.3 monolayer cells for various durations of incubation to evaluate the penetration efficiency of NPs across BBB and their uptake by neuron‐mimic SH‐SY5Y cells in the basolateral chamber (Figure 2C). Interestingly, flow cytometry assessment revealed significant increases in cellular uptake of Cy5‐p(PB)_10_/(TB)_30_ NPs in SH‐SY5Y cells post‐BBB crossing, with 6.1‐fold and 3.9‐fold increases in average fluorescence signal at the initial 3 h than that of the Cy5‐PEG‐PLGA NPs (control group) and Cy5‐p(P)_10_/(TB)_30_ NPs, respectively (Figure 2D). At 12 h, this increase was even more pronounced, reaching 17.3‐fold and 6.5‐fold higher levels than the group treated with Cy5‐PEG‐PLGA‐Cy5 NPs and Cy5‐p(P)_10_/(TB)_30_ NPs, respectively. The mean fluorescence intensity (MFI) of cell uptake results analyzed by confocal microscopy also reflected similar trends in cellular uptake post‐NPs treatment (Figure 2E; Figure S21, Supporting Information). These findings revealed that damaged neuronal cells effectively internalized penetrative NPs that had traversed the BBB, allowing for even faster drug release and effective drug concentration.

Further analysis of cell uptake mechanisms showed that the intervention with amiloride and chlorpromazine (CPZ) led to reductions in cell uptake efficiency of 16.0% and 35.6%, respectively; however, genistein intervention exhibited negligible inhibitory effects on cellular uptake (Figure S22, Supporting Information). Collectively, the findings suggest that the internalization of NPs by cells predominantly is dependent on micropinocytosis and clathrin‐mediated endocytosis mechanisms. Additionally, the efficiency of NPs uptake in neuronal cells after incubation at low temperature (4 °C) reduced by 50.1%, indicating that NPs uptake under physiologically relevant conditions is mediated by an energy‐dependent endocytosis process.

### 2.3 Attenuation of Glutamic Acid‐Induced Neuron Injury

#### 2.3.1 ROS‐Scavenging Effect in Injured Neurons

**Figure 3:**
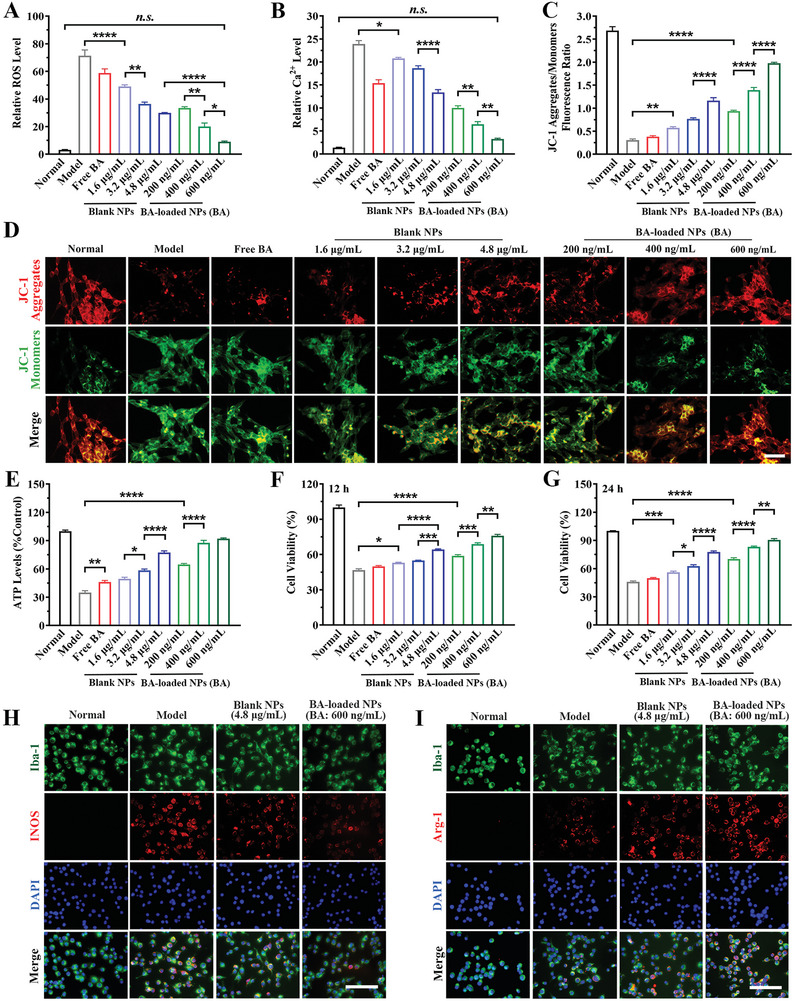
The cytoprotective effects of formulations. A) The quantitative flow cytometry results for intracellular ROS levels in glutamate‐induced injured SH‐SY5Y cells with various treatments. n = 3. B) The quantitative flow cytometry results of intracellular Ca^2+^ levels in a glutamate‐induced excitotoxicity acute neuronal cell injury model (injured SH‐SY5Y cells) with various treatments. n = 3. C) Semi‐quantitative results and D) fluorescence images of JC‐1 aggregate/JC‐1 monomer to represent mitochondrial membrane potential (MMP). n = 6. Scale bar: 50 µm. n = 6. E) The intracellular ATP levels of injured SH‐SY5Y cells after treatment with various formulations. n = 3. F) The cell viability recovery of injured SH‐SY5Y cell following (F) 12 h and G) 24 h of treatments. n = 3. Immunofluorescent results of co‐staining pan‐microglia marker (Iba1) and H) M1‐microglia marker (iNOS) or I) M2‐microglia marker (Arg‐1) for evaluating the M1 or M2 phenotype polarization of microglia (BV2 cells). Scale bar: 100 µm. n = 6. Data are shown as mean ± SEM.

In a glutamate‐induced acute neuronal cell injury model, treatment with blank NPs significantly reduced intracellular ROS levels, with decreases of 31.2%, 48.9%, and 57.9% at polymer concentration of 1.6, 3.2, and 4.8 µg·mL^−1^, respectively. Possible explanations for these reductions include ROS consumption by responsive TK bond cleavage and dissociated borneol‐mediated direct oxygen‐free radical scavenging effect in injured neurons (**Figure** **3**A; Figure S23, Supporting Information). The group receiving BA‐loaded NPs at BA doses of 400 and 600 ng·mL^−1^ (corresponding polymer: 3.2 and 4.8 µg·mL^−1^, respectively) showed stronger inhibitory effects on intracellular ROS, with reductions of 71.8% and 87.3%, respectively, and even returning to baseline ROS levels in healthy cells. The modulation of Ca^2+^ homeostasis by BA might prevent the perturbation of inner mitochondrial membranes, thus averting the continuous generation and release of ROS via the maintenance of mitochondrial respiration rates and the inhibition of key ROS production sites (e.g., the NADPH oxidase enzyme). The regulation of intracellular ion homeostasis could potentially disrupt the intracellular feedback regulatory loop that causes cell injury due to Ca^2+^ overload and ROS overproduction, explaining formulation's superior ROS elimination ability following BA delivery.

#### 2.3.2 Elimination of Calcium Overload in Injured‐Neurons

In injured neurons, free BA (600 ng·mL^−1^) decreased [Ca^2+^]_i_ levels by 35.5%, probably due to its limited water solubility, which restricts its ability to persist in a free drug form within the medium for an extended period (Figure 3B; Figure S24, Supporting Information). High dose of blank NPs (polymer: 4.8 µg·mL^−1^) lowered peak [Ca^2+^]_i_ levels by 44.1%. This Ca^2+^ removal effect was attributed to the ROS scavenging effects of responsively dissociated borneol, which restored the functionality of Ca^2+^ transport proteins in the plasma membrane and organelles that contribute to ion homeostasis. However, its capacity to scavenge the already elevated [Ca^2+^]_i_ was restricted. As expected, BA‐loaded NPs effectively reduced overloaded [Ca^2+^]_i_ by 58.0%, 73.0%, and 86.4% at BA concentrations of 200, 400, and 600 ng·mL^−1^ and corresponding polymer of 1.6, 3.2, and 4.8 µg·mL^−1^ in injured neuronal cells, indicating a positive dose‐effect relationship between the calcium elimination effects and formulation dose within the test range. Gratifyingly, treatment with BA‐loaded NPs at 600 ng·mL^−1^ of BA restored [Ca^2+^]_i_ levels to normal levels in healthy cells. This notable restoration was accomplished by effectively Ca^2+^ chelating with BA after improving water solubility through payload encapsulation, which was further enhanced by the synergistic effect of dissociated borneol on Ca^2+^ elimination, ultimately maximizing the recovery of Ca^2+^ homeostasis.

#### 2.3.3 Recovery of Mitochondrial Function and Cell Viability

The imbalance of [Ca^2+^]_i_ triggered large influx of calcium into the mitochondria, coupled with an excess production of mitochondrial oxidative free radicals, the collapse of the mitochondrial membrane potential (MMP), impaired mitochondrial respiration, and subsequent ATP depletion.^[^ ^17^ ^]^ Blank NPs (polymer: 4.8 µg·mL^−1^) treatment restored MMP in an acute neuronal cell model, which culminated in a 3.0‐fold increase in the MFI ratio of JC‐1 aggregate to monomer, and a 2.2‐fold boost in intracellular ATP levels compared to the model group (Figure 3C–E). BA‐loaded NPs at 600 ng·mL^−1^ BA dosage (corresponding polymer: 4.8 µg·mL^−1^) significantly raised the relative aggregate/monomer MFI ratio by 6.5‐fold and ATP levels by 2.6‐fold than model group. The regulation of Ca^2+^ homeostasis by BA, which prevents the collapse of the mitochondrial membrane, aids in the recovery of mitochondrial functionality, and promotes the restoration of mitochondrial energy metabolism, is likely responsible for this effect. After being treated with blank NPs (polymer: 4.8 µg·mL^−1^), the cell viability went back up from 46.8% to 64.3% and 77.6% after 12 and 24 h, respectively (Figure 3F,G). It is logical that the BA‐loaded NPs treatment at a BA dosage of 600 ng·mL^−1^ was noticeably more effective in rescuing the acutely injured neuronal cells, with cell viability reaching 75.8% at 12 h and even reaching 94.0% at 24 h of incubation.

#### 2.3.4 Promotion of the Microglia Transformation from M1 to M2 Phenotypes In Vitro

Compared to model group, murine microglial BV2 cells treated with blank NPs (polymer: 4.8 µg·mL^−1^) showed a reduction in iNOS (pro‐inflammatory M1 phenotype marker) levels by 58.7% and an increase in Arg‐1 (anti‐inflammatory M2 phenotype marker) expression by 4.9‐fold, indicating that the ROS‐responsively dissociated borneol could resist the LPS‐induced pro‐inflammatory phenotype polarization of microglia (Figure 3H,I; Figures S25 and S26, Supporting Information). It is compelling that BA‐loaded NPs at a middle dose (BA: 400 ng·mL^−1^, polymer: 3.2 µg·mL^−1^) exhibited superior inhibitory effects on microglia polarization toward the iNOS‐positive M1 phenotype (reduced by 72.9%), while simultaneously effectively increasing the Arg‐1‐positive anti‐inflammatory M2 phenotype by 6.5‐fold (Figures S25 and S26, Supporting Information). Most microglia were transformed into an Arg‐1‐positive anti‐inflammatory M2 phenotype at high dosage of BA‐loaded NPs (BA: 600 ng·mL^−1^, polymer: 4.8 µg·mL^−1^), as evidenced by a 7.4‐fold increase in MFI of the anti‐inflammatory phenotype marker (Arg‐1) than model group (Figure 3I).

Accordingly, the as‐prepared formulation promptly and effectively eliminated the initiator of the cell damage cascade, intracellular calcium overload. This resulted in a notable reduction in intracellular oxidative stress, mitochondrial function restoration, and energy metabolism improvement, ultimately rescuing dying cells and restoring cellular “health” status.

### 2.4 In Vivo Borneol‐Mediated Brain Targeting

#### 2.4.1 In Vivo Biodistribution

**Figure 4:**
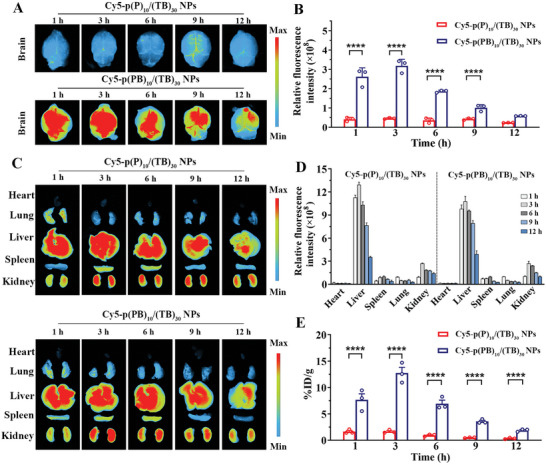
Brain‐targetability and in vivo distribution of p(PB)_10_/(TB)_30_ in the MCAO mice. A) Ex vivo fluorescence image of Cy5‐p(PB)_10_/(TB)_30_ NPs and Cy5‐p(P)_10_/(TB)_30_ NPs in the whole brain. B) The semi‐quantitative fluorescent intensity of Cy5‐p(PB)_10_/(TB)_30_ NPs and Cy5‐p(P)_10_/(TB)_30_ NPs in brains. n = 3. C) Fluorescence imaging of major organs and D) the quantified fluorescence intensity. n = 3. E) The quantitative fluorescent intensity results in the brain tissue homogenate from mice treated with Cy5‐p(PB)_10_/(TB)_30_ NPs and Cy5‐p(P)_10_/(TB)_30_ NPs. ID/g represented percent injected dose per gram of organ, which indicated the localization ratio in brain. n = 3. Data are shown as mean ± SEM.

BA‐loaded NPs demonstrated acceptable hemocompatibility at dosages ranging from 12.5 to 800 µg·mL^−1^ (Figure S27, Supporting Information). A mouse model of middle cerebral artery occlusion (MCAO) was established by inducing 1 h of ischemia followed by reperfusion. To determine their distribution in vivo, different Cy‐5 labeled NPs were administered intravenously (*i.v*.) into MCAO mice at the onset of reperfusion. As shown in **Figure** **4**A,B, only weak fluorescence signals were distributed in the brains of mice treated with Cy5‐p(P)_10_/(TB)_30_ NPs at all time intervals. In contrast, Cy5‐p(PB)_10_/(TB)_30_ NPs rapidly localized in the brains of MCAO mice, with significantly higher cerebral fluorescence levels observed within 1 h (6.2‐fold increase) and peaking at 3 h (8.7‐fold increase) throughout the entire brain compared to the group treated with Cy5‐p(P)_10_/(TB)_30_ NPs. As depicted in Figure 4C,D, the liver fluorescence signal of mice treated with Cy5‐p(PB)_10_/(TB)_30_ NPs was weaker than that of Cy5‐p(P)_10_/(TB)_30_ NPs during the first 1 and 3 h, indicating that brain‐targeted enrichment of Cy5‐p(PB)_10_/(TB)_30_ NPs reduced non‐specific liver accumulation.

#### 2.4.2 Specific Cerebral Accumulation of NPs In Vivo

Most small‐molecule drugs that enter the brain via intravenous delivery have extremely low efficiency, with less than 1% injected dosage per gram of tissue (ID/g), and often as low as 0.1% ID/g.^[^ ^18^ ^]^ Although several recently reported brain‐targeted nanocarriers improve drug biodistribution in the brain, their percent‐injected dose per unit time (1 or 3 h) after intravenous injection remains less than 3% ID/g and rapidly decreases at the following time points.^[^ ^19^ ^]^ Cy5‐p(PB)_10_/(TB)_30_ NPs achieved a high cerebral distribution with up to 7.6% ID/g and 12.7% ID/g of the total injected dose, representing a 4.8‐fold and 7.8‐fold increase compared to the group treated with Cy5‐p(P)_10_/(TB)_30_ NPs at 1 h (1.6% ID/g) and 3 h (1.7% ID/g) in the brains of MCAO mice following single intravenous injection (Figure 4E). The brain accumulation was maintained at 6 and 9 h, with levels that were 7.4‐fold and 7.3‐fold higher than those of the Cy5‐p(P)_10_/(TB)_30_ NPs‐treated group, respectively.

These findings indicated that modifying borneol at the surface region of polymer NPs led to a significant enhancement in the rapid and selective localization as well as prolonged retention of NPs in damaged brain tissues, allowing for better and more efficient transport of the payload into the ischemic region to achieve effective therapeutic drug concentration.

### 2.5 Recovery of Neurological Function in MCAO Mice

**Figure 5:**
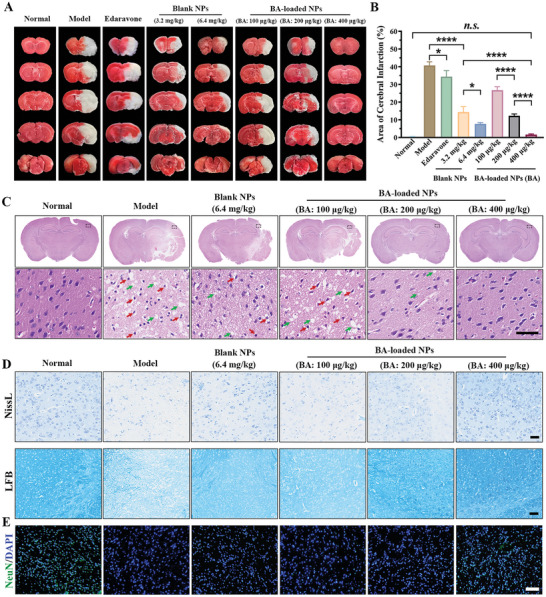
BA‐loaded NPs improved neurological function recovery in MCAO mice. A) The typical TTC staining images of the brain slices of normal mice and MCAO mice with treatments, white indicated infarcted areas, while non‐infarcted areas were red. B) Quantitative results of cerebral infarct volume (ratio of white infarcted areas to the entire cerebral area) in different groups. n = 5. C) H&E staining of the brains from each treatment. Scale bar: 50 µm. Red arrows indicate neuronal shrinkage, and green arrows indicate the regions of vacuolization. D) Representative Nissl staining of brain sections. Scale bar: 50 µm, n = 3. LFB staining of brain sections. Scale bar: 100 µm, n = 3. E) Immune‐fluorescent staining of NeuN of brain tissue sections. Scale bar: 100 µm. Data are shown as mean ± SEM.

Neurological behavior in MCAO mouse models was assessed using the Longa score method, with a higher score denoting more severe damage. As depicted in Figure S28 (Supporting Information), treatment with Edaravone (10 mg·kg^−1^), a commonly used pharmacological agent for cerebral ischemic stroke, resulted in a modest improvement of neurobehavioral function in MCAO mice, yielding a 31.8% decrease in the Longa score. A single dose of blank NPs (polymer: 3.2 mg·kg^−1^) resulted in a 36.3% reduction in the average Longa score in MCAO mice. However, increasing dosage of blank NPs (polymer: 6.4 mg·kg^−1^) only led to an additional 8.3% reduction in the Longa score. Further elevating the blank NPs dosage (polymer: 9.6 mg·kg^−1^) did not further improve the Longa score (data not shown). Treatment with BA‐loaded NPs at BA dosage of 400 µg·kg^−1^ (corresponding polymer: 3.2 mg·kg^−1^) effectively restored neurobehavioral function to a level comparable to that of healthy mice, showing significantly ameliorated neurological deficits and reinstated spontaneous locomotion. As shown in **Figure** **5**A,B, Edaravone (10 mg·kg^−1^) treatment led to a reduction in the brain infract area from 40.8 ± 2.0% (model group) to 34.3 ± 1.6%. Treatment with blank NPs (polymer: 3.2 mg·kg^−1^) resulted in an obvious reduction in the brain infarction volume, reaching to 14.4 ± 1.4% of the total brain infarction volume. Increasing dosage of the blank NPs (polymer: 6.4 mg·kg^−1^) led to the total brain infarction volume declining to 7.6 ± 0.7%. The findings supported that the responsively dissociated borneol treatment reduced infract area to a certain extent, with a similar alleviation trend as the reduction in neurobehavioral scores. The administration of blank NPs (polymer: 9.6 mg·kg^−1^) did not further diminish the brain infarction volume (data not shown), possibly attributed to the borneol's inability to block the initiator of cell damage, which was intracellular calcium overload. Therefore, the group treated with blank NPs (polymer: 6.4 mg·kg^−1^) was used as the control group for subsequent experiments. The BA‐loaded formulation at BA doses of 100, 200, and 400 µg·kg^−1^ (corresponding polymer: 0.8, 1.6, and 3.2 mg·kg^−1^) demonstrated a great protective effect toward the injured ischemic penumbra, drastically reducing infarction volume to 26.8 ± 1.9%, 12.2 ± 1.1%, and 1.7 ± 0.3% of the total brain volume, respectively. By encapsulating BA, the formulation gains the capability to block intracellular upstream pathological injury cascade damage via BA‐mediated calcium elimination. This results in prominent synergistic effects that provide far greater benefits than a single borneol agent.

Single injection of medium‐dose BA‐loaded NPs (BA: 200 µg·kg^−1^) effectively reduced brain tissue injury in MCAO mice after 24 h, as evidenced by alleviated neuronal shrinkage (red arrows) and fewer regions of vacuolization (green arrows) according to H&E‐staining sections (Figure 5C). Increasing the dosage of BA‐loaded NPs (BA: 400 µg·kg^−1^) treatment in MCAO mice further observed that the structure of neuronal cells in the brain tissue sections were arranged evenly, and the intercellular space and cell nuclei returned to their intact state. The neuronal structures and function in mice can be effectively restored to a state that closely resembles that of normal mice by administering BA‐loaded NPs at a BA dosage of 400 µg·kg^−1^, showing noticeable enhancement in the amount of Nissl bodies and a significant reduction in neuron looseness, as well as a complete fiber arrangement without vacuolation (Figure 5D).

It is important to note that the blank NPs treatment (polymer: 6.4 mg·kg^−1^) was able to reverse the loss of neurons, as evidenced by a 2.7‐fold rise in the expressed level of neuron‐specific marker NeuN, in contrast to the minimal expression of NeuN in model group (Figure 5E; Figure S29, Supporting Information). The high‐dose BA‐loaded NPs intervention (BA: 400 µg·kg^−1^, polymer: 3.2 mg·kg^−1^) resulted in a notable recovery of neurons in the cerebral ischemic penumbra, as evidenced by 4.1‐fold increase in the expression abundance of the NeuN. These findings indicated that the formulation treatment effectively and steadily promoted neuronal survival and improved neuronal function, potentially attributing to the synergistic maintenance of ion homeostasis, restoration of redox state, and recovery of organelle function of targeted neuronal cells facilitated by the co‐delivery of borneol and BA.

### 2.6 MRI Brain Image Analysis in MCAO Mice

**Figure 6:**
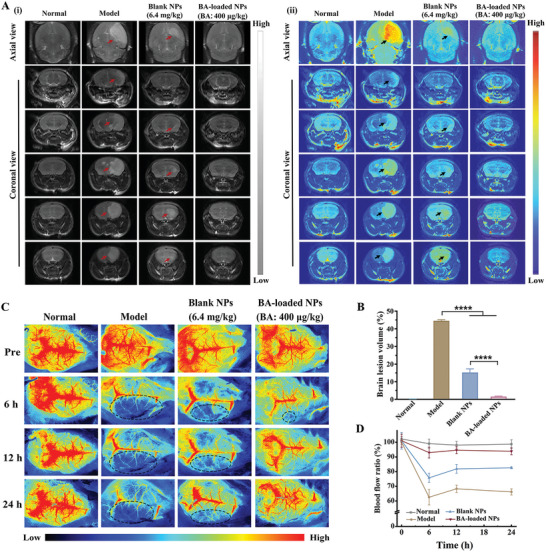
BA‐loaded NPs reduce brain tissue infarction and promote blood flow recovery in MCAO mice. A) (i) T2‐weighted magnetic resonance (MRI) imaging and (ii) their corresponding false color scans. B) the semi‐quantitative infarct volume results (hypersignal area in MRI imaging) of MCAO mice from each group. n = 3. C) Color‐coded laser speckle images that reflect blood flow reperfusion. D) Semi‐quantitative analysis of blood flow ratio at different time points in ischemic lesions. n = 3. Data are shown as mean ± SEM.

The T2‐weighted magnetic resonance imaging (MRI) scans of MCAO mice revealed that treatment with blank NPs (polymer: 6.4 mg·kg^−1^) caused an obvious reduction in cerebral infarct lesion, as indicated by a decreased region (by 65.7%) of cerebral hyperintensity area (highlighted by a white arrow) in both axial views and consecutive coronal views (**Figure** **6**A,B). Notably, the group with treatment of BA‐loaded NPs (BA: 400 µg·kg^−1^, corresponding polymer: 3.2 mg·kg^−1^) exhibited almost no infarction in both axial and coronal views and a substantial decrease in hypersignal area of 96.3%.

### 2.7 Brain Blood Flow Recovery in MCAO Mice

The spatiotemporal changes of ischemic cortical cerebral blood flow (CBF) were evaluated using laser speckle imaging. The penumbral area of MCAO mice administered blank NPs (polymer: 6.4 mg·kg^−1^) witnessed an improvement in microvascular reperfusion over time, with CBF increasing from 66.2 ± 2.1% to 82.6 ± 0.6% at 24 h, indicating partial recanalization of blood vessels (Figure 6C,D). Gratifyingly, BA‐loaded NPs (BA: 400 µg·kg^−1^, polymer: 3.2 mg·kg^−1^) treatment rapidly increased the CBF to 92.9 ± 3.7% within the initial 6 h and maintained the CBF of 93.9 ± 2.4% after 24 h, which suggests a considerably improved blood supply and a prominent efficacy in preventing “no‐reflow” in microvasculature induced by I/R.

### 2.8 Alleviation of Oxidative Stress and Apoptosis in MCAO Mice

**Figure 7:**
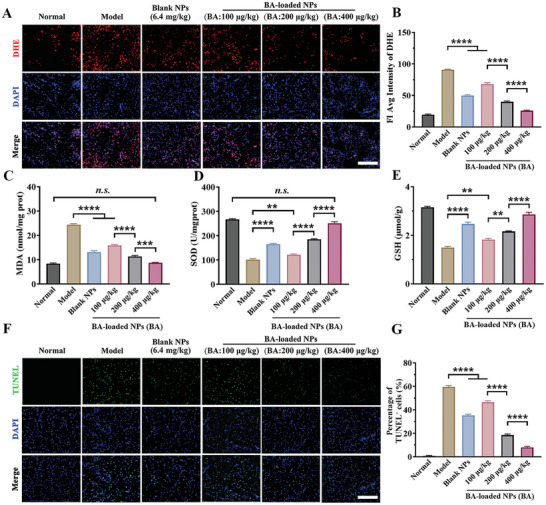
Neuroprotection of BA‐loaded NPs from cerebral I/R injury in MCAO mice. A) DHE staining for evaluating the superoxide in brain of MCAO mice, and B) the semi‐quantitative results. Scale bar: 200 µm. n = 6. C) The MDA, D) SOD, and E) the GSH levels in tissue homogenate. n = 8‐12. F) Representative images of TUNEL staining for evaluating the apoptotic cells, scale bar: 200 µm, and G) the semi‐quantitative results of TUNEL‐positive cell proportion. n = 6. Data are shown as mean ± SEM.

As shown in **Figure** **7**A,B, MCAO mice treated with blank NPs at 6.4 mg·kg^−1^ of polymer exhibited a predominant decrease in superoxide levels (indicated by DHE fluorescent intensity) with a 45.2% reduction in the injured cerebral tissue. Notably, the treatment with BA‐loaded NPs at middle dose (polymer: 1.6 mg·kg^−1^, BA: 200 µg·kg^−1^) and high dose (polymer: 3.2 mg·kg^−1^, BA: 400 µg·kg^−1^) declined the superoxide levels (represented by MFI of DHE) by 56.1% and 77.4% compared to the model group, respectively.

Additionally, the group that received a single dosage of BA‐loaded NPs at 100, 200, and 400 µg·kg^−1^ of BA exhibited substantial inhibition of MDA levels with reductions of 34.7%, 53.3%, and 64.3%, and an effective elevation of SOD levels by 1.2, 1.8, and 2.5 times in brain tissue homogenate of MCAO mice, and was restored to normal levels at 400 µg·kg^−1^ of BA, indicating a remarkable inhibitory impact on the lipid peroxidation process and significant recovery of the antioxidative state (Figure 7C,D). The levels of anti‐oxidative GSH also exhibited a similar trend of increase, in line with the improvement of the antioxidative SOD component following the treatment (Figure 7E).

In Figure 7F,G, treatment with blank NPs (polymer: 6.4 mg·kg^−1^) decreased the proportion of apoptotic cells (indicated by the green TUNEL positive fluorescence signal) in the cerebral ischemic penumbra tissue of MCAO mice from 59.5 ± 1.0% to 35.2 ± 1.1% after 24 h of I/R injury. Even a medium dose of BA‐loaded NPs (BA: 200 µg·kg^−1^, polymer: 1.6 mg·kg^−1^) significantly reversed cell apoptosis, resulting in an overall reduction in TUNEL‐positive cells to 18.5 ± 0.9%. Strikingly, there were negligible green TUNEL‐positive apoptotic cells (7.9 ± 0.9%) visible in the cerebral section after high dose of BA‐loaded NPs treatment (BA: 400 µg·kg^−1^, polymer: 3.2 mg·kg^−1^).

These findings indicate that a single injection of BA‐loaded NPs can facilitate the restoration of neuronal cells redox status in the ischemic penumbra, potentially protecting critical cellular proteins or organelles from the lethal effects of oxidative stress injury, as well as significantly inhibiting cell apoptosis, providing noticeable neuroprotective effects.

### 2.9 Remodeling of Inflammatory Microenvironment in Ischemic Penumbra

**Figure 8:**
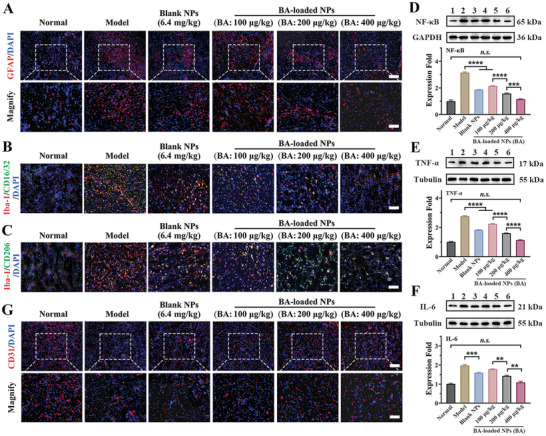
BA‐loaded NPs inhibit the progression of neuroinflammation and modulate the cerebral inflammatory microenvironment in MCAO mice. A) Immunofluorescence staining images of GFAP‐labeled activated astrocytes in cerebral penumbra from MCAO mice with different treatments. n = 5 Scale bar: merge (100 µm), magnify (50 µm). B) Representative immunofluorescent results of pan‐microglia marker (Iba1) and pro‐inflammatory M1‐microglia marker (CD16/32) for evaluating the M1 phenotype polarization of microglia in MCAO mice with different treatments. Scale bar: 50 µm. n = 6. C) Representative immunofluorescent results of pan‐microglia marker (Iba1) and pro‐inflammatory M2‐microglia marker (CD206) for evaluating the M2 phenotype polarization of microglia in MCAO mice with different treatments. Scale bar: 50 µm. n = 6. Protein expression abundance of D) NF‐κB, E) TNF‐*α*, and F) IL‐6 in cerebral tissue homogenate. n = 3. Lane 1, Normal group; Lane 2, Model group, Lane 3, Blank NPs‐treated group; Lane 4, 5, and 6 was BA‐loaded NPs‐treated group at BA dose of 100, 200, and 400 µg·kg^−1^. G) Representative immunofluorescent results of CD31, a biomarker for endothelial cells, for evaluating the recovery of microvessel endothelium in MCAO mice with different treatments. Scale bar: merge (200 µm), magnify (100 µm). n = 6. Data are shown as mean ± SEM.

Injured neurons produce endogenous damage‐associated molecular patterns (DAMPs), which induce microglia and astrocytes to overactivate in the acute phase and release large amounts of inflammatory mediators.^[^ ^20^ ^]^ This process progresses into a pro‐inflammatory microenvironment that damages endothelial cells and disrupts microcirculation, exacerbating neuronal injury. As depicted in **Figures** **8**A and S30 (Supporting Information), the treatment of blank NPs at 6.4 mg·kg^−1^ of polymer led to a 30.1% decline in GFAP‐positive activated astrocytes in the cerebral penumbra area in comparison to model group. The group that was given BA‐loaded NPs at 400 µg·kg^−1^ of BA (polymer: 3.2 mg·kg^−1^) showed a substantial reduction (by 54.6%) of GFAP‐positive astrocytes, and their numbers returned to a level comparable to the resting state, indicating a significant inhibition in astrocyte migration and activation in the brain following I/R injury.

Blank NPs (polymer: 6.4 mg·kg^−1^) treatment were found to inhibit the polarization of Iba1‐positive microglia (activated microglia) toward the CD16/32‐positive M1 phenotype (pro‐inflammatory phenotype) with a 47.8% reduction in the cerebral ischemia penumbra within 24 h when compared to the model group (Figure 8B; Figure S31, Supporting Information). BA‐loaded NPs treatment suppressed the proliferation and activation of Iba1‐positive microglia and decreased the proportion of M1 microglia phenotype by 20.9%, 52.8%, and 72.8% at BA dosages of 100, 200, and 400 mg·kg^−1^ (corresponding polymer: 0.8, 1.6, and 3.2 mg·kg^−1^), respectively. Unsurprisingly, these treatments led to significant increase in anti‐inflammatory CD206‐positive M2 microglia proportions (anti‐inflammatory phenotype) by 2.5‐, 3.5‐, and 4.5‐fold (Figure 8C; Figure S32, Supporting Information).

Additionally, blank NPs (polymer: 6.4 mg·kg^−1^) treatment further inhibited NF‐κB p65 translocation subunits to the cellular nucleus with a reduction of their level by 40.7% and reduced the downstream TNF‐*α* by 33.9% and IL‐6 by 19.3% in MCAO mice, thereby partially initiating a molecular‐level suppression of the inflammation response (Figure 8D–F). Specifically, the levels of NF‐κB, TNF‐*α*, and IL‐6 were diminished by 63.3%, 59.0%, and 44.6% after administration of BA‐loaded NPs at BA doses of 400 µg·kg^−1^ (corresponding polymer: 3.2 mg·kg^−1^) and reduced to levels close to healthy mice.

After treatment with blank NPs (polymer: 6.4 mg·kg^−1^), CD31‐marked microvascular endothelial cells increased significantly, with a 3.2‐fold rise in MFI of expressed CD31 in the cerebral ischemic penumbra of MCAO mice (Figure 8G; Figure S33, Supporting Information). Notably, we observed that administering BA‐loaded NPs at a BA dose of 400 µg·kg^−1^ effectively restored cerebral micro‐vessel density and endothelium, as evidenced by a 5.2‐fold increase in MFI in CD31 in the injured brain, reaching a level almost identical to that of the healthy mice.

The formulation can remodel the inflammatory microenvironment by reducing astrocytes and microglia migration and activation, blocking microglia proliferation and activation, reprogramming microglia into an anti‐inflammatory M2 phenotype, suppressing NF‐κB/TNF‐*α*/IL‐6 signaling in the cerebral ischemic penumbra, and promoting brain micro‐vessel endothelium recovery. This synergistic effect of BA and borneol on neuronal recovery contributed to blocking the feedback loop with the inflammatory cascade and microcirculation disorders, which accelerated brain tissue recovery.

### 2.10 Restoration of the Integrity and Function of the BBB In Vivo

**Figure 9:**
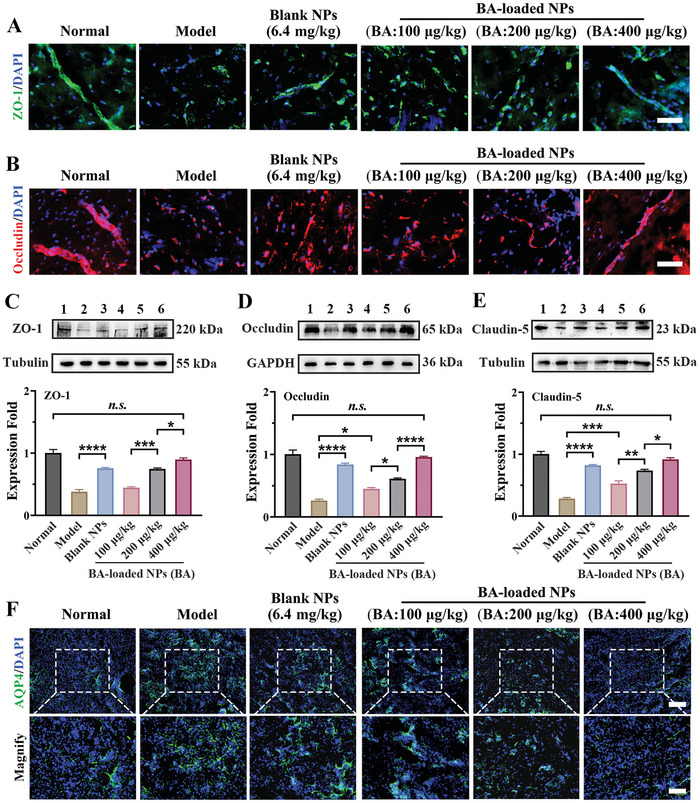
BA‐loaded NPs promote BBB remodeling after stroke. A) Representative immunofluorescent results of key TJs’ complex proteins, ZO‐1, in MCAO mice with different treatments. Scale bar: 20 µm. n = 6. B) Immunofluorescent results of key TJs’ complex proteins, Occudin, in MCAO mice with different treatments. Scale bar: 20 µm. n = 6. The representative western blot analysis of the TJ's complex proteins C) ZO‐1, D) Occludin, and E) Claudin‐5 and their semi‐quantitative results. n = 3. Lane 1, Normal group; Lane 2, Model group, Lane 3, Blank NPs‐treated group; Lane 4, 5, and 6 was BA‐loaded NPs‐treated group at BA dose of 100, 200, and 400 µg·kg^−1^. F) Representative immunofluorescent results of AQP4 in MCAO mice with different treatments. Scale bar: merge (200 µm), magnify (100 µm). n = 6. Data are shown as mean ± SEM.

The TJs proteins (ZO‐1, Occludin, and Claudin‐5) were presented as essential components that preserve the integrity of the BBB during the late stages of reperfusion. **Figure** **9**A,B and Figures S34 and S35 (Supporting Information) show that ZO‐1 and Occludin protein expression fluorescent signals in brains of MCAO mice were weak and discontinuous. However, blank NPs (polymer: 6.4 mg·kg^−1^) increased their expressed levels by 4.0‐fold and 3.6‐fold at the late cerebral reperfusion stage (24 h). The group that received high‐dose BA‐loaded NPs (BA: 400 µg·kg^−1^, polymer: 3.2 mg·kg^−1^) exhibited a robust, intact, continuous, and dense fluorescence signal, with MFI of ZO‐1 and Occludin expression increasing to 4.9‐fold and 4.5‐fold, respectively. Furthermore, ZO‐1 and Occludin protein expression abundance in western blot analysis exhibited similar increased trends in brain tissue following treatment (Figure 9C,D). Furthermore, Claudin‐5, the most enriched component in brain endothelial cells for maintaining BBB integrity, was increased by as much as 2.9‐fold in blank NPs‐treated group (polymer: 6.4 mg·kg^−1^) and increased by 3.2‐fold in BA‐loaded NPs‐treated group at BA dose of 400 µg·kg^−1^ (polymer: 3.2 mg·kg^−1^), respectively (Figure 9E). Accordingly, the responsively dissociated borneol molecules played a critical role in alleviating pathological BBB disruption by directly upregulating the expression levels of TJs’ complex protein while the co‐delivered BA might further synergistically enhance barrier function by inhibiting the endothelium damage via regulating the inflammatory microenvironment in the ischemic penumbra.

Previous studies have identified that a rise in intracellular Ca^2+^ concentration in perivascular astrocytes after brain injury activates calmodulin‐dependent aquaporin 4 (AQP4) and translocates it to the plasma membrane, resulting in cellular edema and brain swelling.^[^ ^21^ ^]^ The 3.4‐fold increase in AQP4 expression in the MCAO model group revealed a severe disruption of the BBB in the brain endothelium (Figure 9F; Figure S36, Supporting Information). The administration of blank NPs (polymer: 6.4 mg·kg^−1^) resulted in a 42.7% reduction in the levels of overexpressed AQP4. During the late reperfusion period (24 h), a high dose of BA‐loaded NPs (BA: 400 µg·kg^−1^, polymer: 3.2 mg·kg^−1^) accelerated BBB recovery, as evidenced by a 66.9% drop in AQP4 level. Thus, BA's pharmacological regulation of calcium homeostasis protected MCAO mice against brain edema by directly impeding the calcium‐dependent postischemic cell‐membrane localization of AQP4. Alternatively, dissociated borneol indirectly inhibits astrocyte activation, resulting in a decrease in AQP4 levels in the later stages of ischemia (refer to Figure 8A). Consequently, the two compounds would undoubtedly complement one another to mitigate the risk of feedback neuronal ischemia and hypoxia.

### 2.11 Inhibitory Effects on Mitochondrial Apoptotic Signaling Pathways

**Figure 10:**
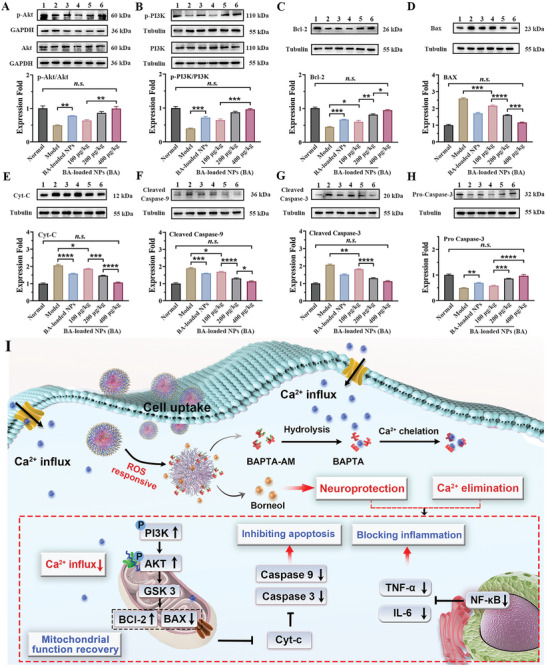
BA‐loaded NPs alleviate the cerebral stroke by inhibiting the mitochondrial apoptosis pathway. The representative western blots and semi‐quantitative results of A) p‐Akt and Akt, B) p‐PI3K and PI3K, C) BCl‐2, D) Bax, E) Cyt‐C, F) Cleaved Capase‐9; G) Cleaved Capase‐3; H) Pro Capase‐3. n = 3. Lane 1, Normal group; Lane 2, Model group, Lane 3, Blank NPs‐treated group; Lane 4, 5, and 6 was BA‐loaded NPs‐treated group at BA dose of 100, 200, and 400 µg·kg^−1^. I) Treatment mechanism diagram of the formulations. Data are shown as mean ± SEM.

After an ischemic stroke, brain tissue repair is closely associated with the activated phosphatidylinositol 3‐kinase (PI3K)/serine‐threonine kinase (Akt) signaling pathway, which acts on downstream target anti‐apoptotic signaling pathways. As shown in **Figure** **10**A,B, administering blank NPs (polymer: 6.4 mg·kg^−1^) resulted in a 1.5‐fold increase in phosphorylated Akt (p‐Akt) and a 1.8‐fold increase in the levels of phosphorylated PI3K (p‐PI3K). BA‐loaded NPs treatment group (BA: 400 µg·kg^−1^, polymer: 3.2 mg·kg^−1^) exhibited 2.0‐fold and 2.6‐fold increase in p‐Akt and p‐PI3K levels, respectively, with levels approaching normalcy.

Figure 10C,D demonstrate that BA‐loaded NPs treatment at BA doses of 100 and 200 µg·kg^−1^ elevated the expression of anti‐apoptotic Bcl‐2 protein, a crucial downstream effector of the p‐Akt/p‐PI3K signaling pathway, by 1.4 and 1.8 times, respectively. In contrast, the expression of Bax protein declined by 16.5% and 38.3%, respectively. When BA‐loaded NPs were administered (BA: 400 µg·kg^−1^, polymer: 3.2 mg·kg^−1^), there was a significant upregulation of Bcl‐2 by 2.1‐fold and a downregulation of Bax by 55.3%. The translocated Bax acts as a channel‐forming protein and causes significant disruption of mitochondrial integrity and permeabilization of the mitochondrial outer membrane, which causes substantial releases of Cyt‐C (increased by 2.0‐fold) into the cytosolic compartment of neurons in MCAO mice. It is inspiring that the amount of Cyt‐C released dropped by 48.3% in the brains of MCAO mice that were administrating high dosage of BA‐loaded NPs (BA: 400 µg·kg^−1^, polymer: 3.2 mg·kg^−1^) and eventually returned to normal levels (Figure 10E).

BA‐loaded NPs downregulated the key downstream apoptosis‐inducing factors, as indicated by reduced caspase‐9 levels by 10.7%, 31.3%, and 40.4% and declined caspase‐3 levels by 12.0%, 37.0%, and 45.4% at BA doses of 100, 200, and 400 µg·kg^−1^ (corresponding polymer: 0.8, 1.6, and 3.2 mg·kg^−1^) (Figure 10F,G). In particular, the highest dose nearly restored their levels to those of healthy mice. Conversely, pro‐caspase‐3 exhibited an opposite trend (Figure 10H). The impact of blank NPs (polymer: 6.4 mg·kg^−1^) on downregulating activated caspase‐9 and caspase‐3 was not pronounced (declined by 15.5% and 26.7%, respectively).

These findings revealed that treatment with BA‐loaded NPs strongly activated p‐PI3K/p‐Akt signaling and regulated the downstream Bcl‐2/Bax/Cyto‐C/caspase3,9 signaling pathway (Figure 10I). The process reflected the ROS‐responsively dissociated borneol of blank NPs, which partially impeded mitochondrial apoptosis due to the rapid scavenging of oxidative free radicals, inhibition of the collapse of mitochondrial membrane potential, and restoration of energy supply, among others. It further underscored the importance of co‐delivered BA‐mediated calcium elimination in preserving mitochondrial dynamics, attenuating mitochondrial dysfunction, and suppressing the cell mitochondrial apoptosis cascade. This dual mechanism ultimately prevented neuronal cell death and effectively reversed ischemic penumbra injury in MCAO mice.

### 2.12 Biosafety Evaluation

As depicted in Figures S35 and S36 (Supporting Information), blood biochemistry markers reflecting liver functionality (AST, ALT) and kidney functionality (BUN, and Cre), as well as the histopathology (representative H&E staining) of major organs (heart, liver, spleen, lung, and kidney) displayed minimal variations between groups treated with different formulations and the control group. These findings collectively indicated a high level of biocompatibility for the dosage of the formulations employed in this study.

## 3 Discussion

In summary, we constructed the borneol‐based copolymer (polymer prodrug) by utilizing the traditional Chinese medicine agent borneol as a functional block, which served as the surface brain‐targeting ligand, inner hydrophobic core for efficient drug loading, or neuroprotective agent. Using the FNC approach, the amphiphilic copolymer self‐assembled into polymer prodrug NPs for encapsulating BAPTA‐AM by kinetically controlling the assembly process in a well‐defined and ordered manner. The optimized formulation exhibited a small particle size (≈43 nm), narrow size distribution (PDI: ≈0.210), high drug encapsulation efficiency (≈86.5%), and great colloidal and storage stability in the form of NPs suspension (stable for over 7 days). Also, the ROS‐responsive BA‐loaded NPs had proven to improve control over the drug release kinetics, enabling prolonged circulation with minimal drug leakage (8.3% at 1 h and 19.0% at 24 h) in the physiological microenvironment, as well as rapid and precise drug release in the pathological microenvironment of the lesion core (reaching 54.8% and 78.5% of the cumulative drug within the first 1 h and 12 h, respectively).

In an MCAO mouse model, these as‐prepared polymer prodrug nanocarriers rapidly traversed the BBB and localized in the cerebral ischemic penumbra, with significantly enhanced brain biodistribution, reaching up to 7.6% ID/g and 12.7% ID/g of the total administered dose at 1 h and 3 h following intravenous injection, respectively, addressing the current status quo of very poor biodistribution in the brain commonly reported intracerebral drug delivery system (typically less than 1% ID/g, and part of them was between 1% and 3% ID/g). The borneol‐based polymer prodrug carriers could be a versatile and universal brain‐targeted delivery system that can be applied to a variety of severe brain diseases to effectively deliver therapeutics that cross the BBB and accumulate in the injured brain to maintain effective blood concentrations at the lesion core.

Following a single dose of BA‐loaded NPs (polymer: 3.2 mg·kg^−1^, BA: 400 µg·kg^−1^), the p‐PI3K/p‐Akt/Bcl‐2/Bax/Cyto‐C/caspase 3,9 mitochondrial apoptosis signaling pathway was mechanically regulated by the responsively dissociated borneol and released BAPTA‐AM through synchronized NPs disassembly, effectively rescuing apoptotic neurons (TUNEL‐positive cell proportion reduced by 51.6%) in MCAO mice's cerebral ischemic penumbra. Furthermore, it efficiently restored the redox status by lowering the oxidative stress component of superoxide (DHE reduced by 77.7%) and lipid peroxidation products (MDA declined by 64.3%), as well as restoring anti‐oxidative species (SOD and GSH) to normal levels. Accordingly, it further remodeled the inflammatory microenvironment in cerebral ischemic penumbra by inhibiting astrocyte activation (GFAP reduced by 54.6%), reprograming microglia polarization via reducing the pro‐inflammatory M1 phenotype by 72.8% and enhancing the anti‐inflammatory M2 phenotype by 4.5‐fold, and blocking the NF‐κB/TNF‐*α*/IL‐6 signaling pathways. The nanoformulation's intervention mechanism successfully restored blood flow reperfusion in the ischemic penumbra from 66.1% to 92.9% at 6 h post‐injection and even recovered to ≈100% at 24 h, significantly reducing the cerebral infarction area by up to 96.1%, ameliorating the histopathology of brain tissue, and improving the neurological behavior of MCAO mice. It also restored the integrity of TJs (increased the expression abundance of ZO‐1, Occludin, and Claudin‐5 by 4.3‐fold, 4.1‐fold, and 3.2‐fold, respectively), facilitated BBB endothelial repair (CD31 increased by 5.2‐fold), and prevented the occurrence of cerebral edema (AQP4 dropped by 66.9%) in the late stage of reperfusion.

In particular, the single injection of the formulation greatly helps to avoid the potential adverse effects of borneol in vivo, as the corresponding dose of contained borneol (0.6 mg·kg^−1^) in the polymer at the optimal dosage of formulation is significantly lower than the borneol administration doses (>200 mg·kg^−1^) used in previous preclinical studies. Moreover, this treatment strategy breaks the current situation of clinical use of borneol in traditional Chinese medicine for prophylactic intervention in CIS, showcasing the potential to be used as a first aid in‐hospital medicine approach for clinical CIS management.

This injectable borneol‐based nanoformulation integrates the functions of “BBB opening”, “calcium homeostasis regulation”, and “neuroprotective effects”, providing multi‐stage sequential therapy for I/R injury as well as comprehensive and systematic brain injury rescue strategies in CIS. Furthermore, the formulation's manufacturing process is facile, continuous, and reproducible, allowing for large‐scale production and addressing the critical barrier in translating the biomanufacturing of nanotherapeutics for clinical use.

## 4 Experimental Section

### Materials

3‐Mercaptopropionic acid, trifluoroacetic acid (TFA), 4‐dimethylaminopyridine (DMAP), N, N'‐carbonyldiimidazole (CDI), polyethylene glycol (average Mn = 1000) (PEG_1k_), 1‐Hydroxybenzotriazole (HOBT), BAPTA‐AM and were procured from Aladdin. Borneol was obtained from Macklin. Cy5 amine (A8143) and FITC goat anti‐rabbit antibody (K1203) acquired from APExBIO (Houston, USA). mPEG_2k_‐b‐PLGA_75k_ was sourced from Ruixi Biotechnology Co., Ltd. Fluo‐4 AM probe and TUNEL detection kits were purchased from KeyGEN Biotech Co., Ltd. (Nanjing, China). The BCA protein assay kit was purchased from Beyotime Co., Ltd. CCK‐8 (C6005) was purchased from New Cell Molecular Biotech Co., Ltd. Trans‐well chamber culture plate and DMEM medium was purchased from NEST Biotechnology Co. Ltd. The JC‐1 Mitochondrial Membrane Potential Assay Kit (CA0201) was purchased from Shanghai Cytoch Biotechnology Co., Ltd. The TTC dye, MDA and BUN assay kits were acquired from Solarbio Science & Technology Co., Ltd. ATP detection and GSH kits purchased from Beijing Boxbio Science & Technology Co., Ltd. CD31‐specific antibodies (M1511‐8) were purchased from HUABIO. Anti‐Cytochrome C (bsm‐33193M) and anti‐phospho‐Akt antibodies (bsm‐33281M) were purchased from Bioss Co., Ltd (Beijing, China). Anti‐Iba1, CD16/32, CD206, and GFAP antibodies were purchased from Abcam. ZO‐1 (61‐7300) and Occludin (71‐1500) were obtained from Thermo Scientific Invitrogen. Color protein markers were purchased from Shanghai Epizyme. Anti‐NeuN, NF‐κB, Bcl‐2, Bax, caspase‐3 antibodies were purchased from Wanleibio Inc. (Beijing, China). TNF‐*α* (bs‐10802R) and p‐PI3K (bs‐6417R) antibodies was purchased from Bioss Co., Ltd. Fetal bovine serum was sourced from Sperikon Life Science & Biotechnology co., ltd. SOD, CRE, AST, ALT kits were purchased from the Jiancheng Institute of Biotechnology.

### Synthesis and Characterization of Polymer—Synthesis of p(PB)_10_/(TB)_30_ and p(P)_10_/(TB)_30_ Polymer

The synthesis procedures of TK, MA‐TK, MA‐TK‐Borneol, MA‐PEG, MA‐PEG‐borneol were shown in supplementary materials.

The synthesis of polymer prodrug (p(PB)_10_/(TB)_30_) through RAFT polymerization involves the following steps: Initially, MA‐TK‐Borneol monomer (300 mg, 0.6 mmol, 30 eq) and MA‐PEG‐Borneol (252.4 mg, 0.2 mmol, 10 eq) monomer were dissolved in DMF along with (4‐cyano‐4‐(thiobenzoylthio)pentanoic acid) (CPADB) (5.6 mg, 0.02 mmol, 1 eq) and AIBN (0.3 mg, 0.002 mmol, 0.1 eq) in Schlenk vials with continuous stirring, and solution was degassed using three freeze‐pump‐thaw cycles. After 18 h of reaction at 75 °C under anaerobic conditions, the final p(PB)_10_/(TB)_30_ polymer product was obtained by triple precipitation with excess diethyl ether.

The control p(P)_10_/(TB)_30_ polymer was obtained by RAFT polymerization of MA‐TK‐Borneol (MTB, 300 mg, 0.6 mmol, 30 eq) monomer and MA‐PEG (252.4 mg, 0.2 mmol, 10 eq) monomer following the same procedure.

### Synthesis and Characterization of Polymer—Characterization of Polymer

^1^H NMR spectra were recorded on a Bruker AVANCE III 600 MHz NMR spectrometer. GPC was conducted with an Agilent PL gel 5 µm MIXED‐C column manufactured in the UK. The eluent used was 0.02 M LiBr dissolved in dimethyl sulfoxide, with a flow rate of mL·min^−1^, and an operating temperature of 35 °C.

### Preparation of NPs

FNC was exploited to produce NPs. The mixture of polymer and BA was dissolved in a DMSO solution and fed into one inlet (organic phase) of a Confined Impinging Jets (CIJs) mixer device with three channels, while water was simultaneously introduced through the remaining two inlets (aqueous phase). When the ratio of organic phase (O) to water phase (W) was fixed at 1:9, the flow rate and feeding BA concentration was fine‐tuned within the range of 0.2 to 40 mL·min^−1^ and 0.25‐1.5 mg·mL^−1^ for optimization purposes, respectively. The collected samples underwent dialysis against water to remove the remaining DMSO. To create blank NPs, the co‐polymer was introduced through inlet 1, while maintaining all other preparation conditions unchanged. Furthermore, the Cy5‐labelled were prepared by feeding the corresponding co‐polymer into inlet 1 of the CIJ mixer upon the O to W fixed at 1:9, the flow rate fixed at 20 mL·min^−1^. The collected samples underwent dialysis against water to remove the remaining DMSO.

### Characterization of NPs—Particle Size, Surface Charge and Morphology Evaluation

The size distribution and ζ‐potential of NPs were analyzed using Malvern Zetasizer Instruments (ZEN3600, the United Kingdom). The morphology of both nanoparticles was examined with a transmission electron microscope operating at 80 kV.

### Synthesis and Characterization of Polymer—Characterization of NPs—EE and LC Evaluation

The NPs suspension underwent purification through a G50 gel column to separate un‐encapsulated free BA from BA‐loaded NPs, followed by being lyophilized and dispersed in a DMSO solution. Drug concentrations within the NPs were determined using HPLC with a C18 column at 256 nm and a mobile phase of acetonitrile/water (3:2, v/v) containing 0.1% trifluoroacetic acid. All experiments were carried out in triplicate. The EE and LC were calculated using the following formulas:

(1) $$\mathrm{EE}=\left(1-\frac{\left[\mathrm{amount}\;\mathrm{of}\;\mathrm{free}\;\mathrm{drug}\right]}{\left[\mathrm{total}\;\mathrm{amount}\;\mathrm{of}\;\mathrm{drug}\right]}\right)\times 100\%$$

(2) $$\mathrm{LC}=\left(\frac{\left[\mathrm{weight}\;\mathrm{of}\;\mathrm{encapsulated}\;\mathrm{drug}\right]}{\left[\mathrm{total}\;\mathrm{weight}\;\mathrm{of}\;\mathrm{nanoparticles}\right]}\right)\times 100\%$$

### Characterization of NPs—Stability Evaluation of NPs Suspension

To determine the storage stability of NPs, the NPs were incubated in water at 25 °C for 7 days and their particle size and PDI were determined by DLS.

### Characterization of NPs—ROS Responsiveness of NPs

Polymer and BA‐loaded NPs were incubated in PBS medium (10 mM, pH 6.5) and PBS medium (10 mM, pH 6.5) containing 0.5 mM H_2_O_2_ at 37 °C for different times. The structural changes of the polymer were subsequently determined by ^1^H NMR analysis to evaluate the degree of responsiveness to ROS. “Tyndall light” path was recorded by direct photography, while particle size and morphological changes of the NPs were then detected by DLS and TEM.

### Characterization of NPs—In Vitro Release Profile Assessment

NPs were dispersed in PBS (10 mM, pH 6.5 or 7.4) medium and a PBS (10 mM, pH 6.5) solution containing 500 µM H_2_O_2_, and samples were collected at specific times. After being separated by Sephadex G‐50, the concentration of released drug release was determined by HPLC. The released drugs were calculated based on following formula:

(3) $$\mathrm{Drug}\;\mathrm{release}\;\left(\%\right)=\frac{\left[\mathrm{weight}\;\mathrm{of}\;\mathrm{released}\;\mathrm{BA}\right]}{\left[\mathrm{weight}\;\mathrm{of}\;\mathrm{total}\;\mathrm{loaded}\;\mathrm{BA}]\right]}\times 100\%$$

### Cell Study—Cell Culture

SH‐SY5Y and bEnd.3 cells and BV2 microglia cells (Pricella Life Science&Technology Co., Ltd.) were cultured in a cell culture flask containing DMEM medium (NEST Biotechnology) containing 10% (v/v) FBS and 1% (v/v) penicillin and streptomycin in an incubator containing 5% CO_2_ at 37 °C.

### Cell Study—Cytotoxicity Study

SH‐SY5Y cells were seeded in 96‐well plates and cultured overnight. Cells were then added to a basic medium containing BA‐loaded NPs at various concentrations (6.25–800 µg·mL^−1^) and incubated for 24 h after removing mediums. The cell viability was determined according to standard protocols for CCK8 kits.

### Cell Study—Construction of an In Vitro BBB Transwell Model

bEnd.3 cells were seeded in the apical side of the transwell chamber (NEST Biotechnology Co. Ltd.) and the cells were grown for several days to form a confluent monolayer, while their TEER was measured daily by a Millicell‐ERS voltmeter. The TEER value of the monolayer needed to exceed 100 Ω cm^−2^ before the transwell chamber could be used for an in vitro BBB penetration assay. For the assessment of drug permeability, SH‐SY5Y cells were cultured in lower chambers and allowed to grow for 24 h to stimulate neurons.

### Cell Study—Visualization of the Morphological Transformation of Tight Junctions

BBB‐mimic bEnd.3 monolayer cells were treated with p(P)_10_/(TB)_30_ NPs and p(PB)_10_/(TB)_30_ NPs. At predetermined time points, after removing the medium, the cells were fixed by 4% cold paraformaldehyde, permeabilized by 0.1% Triton X‐100/PBS and blocked by a blocking solution with 8% donkey serum. After being incubated with anti‐occludin and anti‐ZO‐1 antibodies at 4 °C overnight, the cells were incubated with secondary antibody at 37 °C (1 h) and then stained with DAPI. The stained cells were examined using an inverted fluorescence microscope.

### Cell Study—Transport Study in the Constructed In Vitro BBB Cell Model

The Cy5‐p(PB)_10_/(TB)_30_ NPs and Cy5‐p(P)_10_/(TB)_30_ NPs were added into the apical chamber, respectively, and incubated with bEnd.3 cell monolayer for varying durations of 1, 3, 6, and 12 h. At each time point, the fluorescence signals from the SH‐SY5Y cells on the basolateral side were detected by flow cytometry (BD Accuri C6, USA) or a CLSM to assess the internalization of NPs by neuron cells after crossing the BBB‐mimic monolayer cells. The cell nuclei were stained with DAPI. Relative MFI was analyzed and semi‐quantified by Flow Jo or Image J software.

### Cell Study—Evaluation of the Cellular Uptake Mechanism

To evaluate the potential cellular uptake mechanisms, the SH‐SY5Y cells were incubated with fresh basal cell medium containing various inhibitors, including macropinocytosis‐dependent endocytosis inhibitor (amiloride, 20 µg·mL^−1^), clathrin‐mediated endocytosis inhibitor (chlorpromazine, CPZ, 1 µg·mL^−1^), caveolae‐mediated endocytosis inhibitor (genistein, 50 µg·mL^−1^). After 1 h of incubation at 37 °C, the cells were washed with PBS and added to fresh basal cell medium containing both different inhibitors and Cy5‐labelled p(BP)_10_/(TB)_30_ NPs for an additional 3 h of incubation. Thereafter, the cells were collected and washed three times with PBS, and the cell uptake efficiency was analyzed using flow cytometry.

In a separate experiment, the cells were pre‐incubated at low temperature (4 °C) for 1 h, treated with Cy5‐labelled p(BP)_10_/(TB)_30_ NPs for 3 h, and then collected and washed three times with PBS, after which the cell uptake efficiency was analyzed using flow cytometry.

### Cell Study—Establishment of a Glutamate‐Induced Acute Neuronal Cell Injury Model and Experiment Groups Setup

SH‐SY5Y cells were seeded in 12‐well plates or 96‐well plates and then cultured overnight and then incubated with a basic medium containing L‐glutamic acid (25 mM) for 6 h to induce the neuronal cell injury model. After removing medium, cells were treated free BA, blank NPs with polymer concentrations of 1.6, 3.2 and 4.8 µg·mL^−1^, as well as BA‐loaded NPs with 200, 400 and 600 ng·mL^−1^ of BA, respectively. The cells incubated with PBS was taken as control group.

### Cell Study—Evaluation of Intracellular Calcium Levels and ROS Levels

Glutamate‐induced injured neuronal cells were treated with different formulations for 12 h. After removing the medium, the cells were treated with a Fluo‐4 AM fluorescent probe (2 mmol·L^−1^) or a DCFH‐DA fluorescent probe (1 mmol·L^−1^) for 30 min at 37 °C in the absence of light, respectively. This was followed by sterile PBS washes, after which cells were separately harvested, and the levels of calcium or ROS were evaluated using flow cytometry, respectively.

### Cell Study—Determination of Mitochondrial Membrane Potential and ATP Content

Glutamate‐induced injured neuronal cells were seeded in 20‐mm glass‐bottom dish and incubated with different formulations for 12 h. Following removal of the medium, an equal volume of JC‐1 staining solution was added to the medium and incubated at 37 °C (30 min). Cells were washed three times, and fluorescence was observed by a fluorescence microscope. Lastly, semi‐quantitative analysis of the MFI of JC‐1 aggregates and JC‐1 monomers was conducted using Image J software. Besides, cells were collected and disrupted by lysis buffer, followed by a centrifugation step at 4 °C to collect supernatants for determining the intracellular ATP level by ATP assay kits.

### Cell Study—Cell Viability Recovery

Glutamate‐induced injured neuronal cells were treated with different formulations for 12 h and 24 h. The cell viability was evaluated by the CCK‐8 assay according to the manufacturer's instructions.

### Cell Study—In Vitro Microglia Polarization Evaluation

BV2 cells were stimulated with 200 µg·mL^−1^ of LPS and treated with different formulations. After 24 h, the cells were fixed with 4% paraformaldehyde, blocked with 5% BSA, and then incubated overnight at 4 °C with the primary antibodies. Afterwards, cells were incubated with the secondary antibody at 37 °C (1 h) and then stained with DAPI. The stained cells were examined using an inverted fluorescence microscope.

### Animal Study—Animals

ICR mice (male, 6 weeks) were fed in constant humidity and temperature with adequate food and water. All animal experiments were conducted following a robust ethical review and in accordance with the ethical guidelines of the Experimental Animal Management Committee of Ocean University of China (OUC‐AE‐2024‐133).

### Animal Study—Hemocompatibility Test

Red blood cells (RBCs) were collected and washed with PBS. 100 µL of the 5% (v/v) RBC suspension was added to each well and then incubated with equal volume NPs at various final concentrations (12.5–800 µg·mL^−1^). RBC suspension added with PBS and Triton X‐100 was taken as a negative control and a positive control, respectively. The absorbance at 576 nm was measured using a microplate reader. Hemolysis percentage was determined using the following formula:

(4) $$\begin{array}{ccc} & & \mathrm{Hemolysis}\;\mathrm{rate}\;\left(\%\right)\\ & & =\frac{\left[A_{576}\;\mathrm{of}\;\mathrm{NPs}\;\mathrm{samples}-A_{576}\;\mathrm{of}\;\mathrm{negative}\;\mathrm{control}\right]}{\left[A_{576}\;\mathrm{of}\;\mathrm{positive}\;\mathrm{control}-A_{576}\;\mathrm{of}\;\mathrm{negative}\;\mathrm{control}\right]}\times 100\% \end{array}$$

### Animal Study—Animal Model Establishment

Mice were anesthetized and placed on constant‐temperature heated operating table set at 37 ± 1 °C. Following standard skin preparation and disinfection procedures, an incision was made along one side of the neck and the tissue was bluntly separated. After careful separation of the left external carotid artery (ECA), internal carotid artery (ICA), and carotid artery (CCA), the distal ends of the ECA and CCA were ligated, and the ICA was clipped. A silicone coated filament (suture plug) was then inserted through an oblique incision in the ligation site of the CCA. The microvascular clip on the ICA was released, allowing suture plug to be placed into the ICA at 5 mm distal to the carotid bifurcation to occlude the blood supply of the middle cerebral artery for 1 h. Following this, the suture plug was slowly withdrawn, and the incision was ligated to construct the MCAO model. Similar surgery was performed without inserting suture plugs for sham‐operated mice.

### Animal Study—Analysis of In Vivo Biodistribution and Percent‐Injected Dose in Brain

Each group of MCAO mice was intravenous injected with Cy5‐p(P)_10_/(TB)_30_ NPs and Cy5‐p(PB)_10_/(TB)_30_ NPs through the tail vein. Euthanasia was performed at 1, 3‐, 6‐, 9‐ and 12‐h post‐injection for ex vivo fluorescent distribution of major organs via a fluorescence imaging system (LumiFluor AVIS T II, Koreshine Faye (Beijing) Technology Co., Ltd) and semi‐quantified using Image J. Besides, brain tissue was homogenized with lysis buffer, centrifuged, and the supernatant was taken. Afterwards, the supernatant was added to methanol in a 1:1 volume ratio to precipitate proteins. After being centrifuged, the supernatant was obtained for analyzing the fluorescence intensity in brain tissue with a UV spectrophotometer. Formulation concentration in supernatant was calculated according to standard curve. The percent‐injected dose in brain quantified as % ID per g brain tissue (organ weight) were calculated using the following formula:

(5) $$\begin{array}{ccc} & & \mathrm{Percent}-\mathrm{injected}\;\mathrm{dose}\;\mathrm{in}\;\mathrm{brain}\;\left(\%\mathrm{ID}/g\right)\\ & & =\frac{\left[\mathrm{measured}\;\mathrm{formulation}\;\mathrm{dose}\;\mathrm{per}\;\mathrm{brian}\;\mathrm{tisse}\right]}{\left[\mathrm{total}\;\mathrm{injected}\;\mathrm{dose}\right]}\times 100\% \end{array}$$

### Animal Study—Experiment Group Setup

The MCAO mice were divided into five groups: model group, a blank NPs group (polymer: 3.2 and 6.4 mg·kg^−1^), and low‐dose BA‐loaded NPs (BA: 100 µg·kg^−1^, polymer: 0.8 mg·kg^−1^), middle‐dose BA‐loaded NPs (BA: 200 µg·kg^−1^, polymer: 1.6 mg·kg^−1^), and high‐dose BA‐loaded NPs (BA: 400 µg·kg^−1^, polymer: 3.2 mg·kg^−1^) groups, each consisting of 12 mice. The sham group was taken as the control group, consisting of 8 mice. Following 24 h of treatment, blood samples were collected, and the mice were euthanized. Brain tissue was then harvested and fixed in 4% paraformaldehyde for histopathological evaluation. Other fresh brain tissue was harvested and stored at ‐80 °C for further use.

Besides, the MCAO mice were intravenously administered Edaravone (10 mg·kg^−1^) at the onset of reperfusion, designating this group as the positive drug treatment group. After assessing the neurobehavioral deficits in the Edaravone‐treated MCAO mice, these subjects were euthanized, and their brain tissue (n = 5) was sliced into 2‐mm sections and stained with TTC dye at 37 °C for 20 min. The sections were then fixed with paraformaldehyde, and the infarct sizes were analyzed using Image J.

### Animal Study—MRI Imaging

After 12 h of reperfusion, T2‐weighted coronal magnetic resonance imaging of the brain was performed using a 3 T MR scanner (Discovery MR 750, GE Medical Systems, USA) with an 8‐channel animal phased‐array coil. Seventeen contiguous coronal images were obtained for each mouse. The imaging parameters included the following: FSE T2, slice thickness of 1.0 mm, TR of 3000 ms, TE of 85.0 ms.

### Animal Study—Neurobehavioral Assessment and Brain Infarction Investigation In Vivo

The neurobehavioral deficit was scored according to Longa’ s five‐point scale based on specific symptoms: 0 points for no symptoms; 1 point for inability to fully extend the front paw symmetrically, 2 points for circling to the opposite side, 3 points for leaning to the opposite side, and 4 points for inability to walk spontaneously. A higher score indicates a more severe behavioral disorder in the animal. Brain tissue (n = 5) was sliced into 2‐mm sections and stained with TTC dye at 37 °C for 20 min. The sections were then fixed with paraformaldehyde and the infarct sizes were analyzed using Image J.

### Animal Study—Histopathological Evaluation

The brain tissue was sliced into 5‐µm sections and stained with H&E, Nissl, and LFB dye according to standard procedures, respectively, and then recorded using the Vectra automated quantitative pathology imaging system from PerkinElmer for examining brain tissue histopathology.

### Animal Study—Blood Flow Reperfusion Assessment

The cerebral blood flow changes in stroke‐affected brain region were assessed using a laser perfusion imager at specific time points (pre‐ischemia, 6‐, 12‐, and 24‐h post‐ischemia) following various treatments. A saline solution was applied to the surface of the cranial window during imaging to preserve the surface moisture, ensuring clear imaging and accurate measurements. The imaging parameters included the following: gain: 185, exposure time: 20 ms, frame rate: 0.03 fps, background threshold: 10, position distance to the thinned cranial window: 240 mm.

### Animal Study—Antioxidant Activity Evaluation In Vivo

The fresh brain tissue was immersed in OCT medium, frozen, sectioned into 5‐µm slices, and then stained with a DHE probe (1 mmol·L^−1^) for evaluating the superoxide. Afterward, the tissue sections were stained with DAPI for labelling the nuclei and imaged with an inverted fluorescence microscope. Additionally, the MDA, SOD, and GSH levels were evaluated in brain tissue homogenates according to standard procedures, respectively.

### Animal Study—TUNEL Assay

Brian frozen sections (5‐µm) were fixed by 4% paraformaldehyde at 25 °C for 30 min and permeabilized by proteinase K for 5 min. Afterwards, TUNEL labelling was executed following the manufacturer's instructions and counter‐staining with DAPI. Six random areas in each sample were chosen to represent the quantity of TUNEL‐positive cells by Image J software.

### Animal Study—Immunofluorescence Staining

The brain frozen sections (5‐µm) were fixed with 4% paraformaldehyde (30 min). They were then incubated with a blocking solution (PBS medium containing 5% BSA and 5% bovine serum) for 2 h. After the washing steps, the slices were incubated with primary antibody overnight at 4 °C, such as anti‐CD31, NeuN, Iba1, CD206, CD16/32, GFAP, and AQP4. Following washing with PBS, slices or cells were incubated at room temperature for 1 h with corresponding secondary antibodies. Fluorescence images were captured with fluorescence microscope and then MFI was semi‐quantitatively analyzed using Image J software.

### Animal Study—Western Blot Analysis

To analyze total protein in brain tissue, lysed samples were separated by 10% SDS‐PAGE gel electrophoresis and transferred to a PVDF membrane. After blocking with skim milk for 2 h, the membranes were probed with various primary antibodies (ZO‐1, Occludin, Claudin‐5, p‐Akt, Akt, p‐PI3k, PI3K, Bcl‐2, Bax, Cyt‐c, Cleaved, Capase‐3, Pro‐Capase‐3, and Cleaved Capase‐9) overnight at 4 °C. β‐tubulin and GAPDH served as the loading controls. Following incubation with an HRP‐conjugated secondary antibody for 1 h at room temperature, the immunoreactive bands were visualized using ECL Western blotting substrate with a Tanon‐5200 Multi system. Protein band intensity was analyzed using ImageJ software, based on data from three independent experiments.

### Animal Study—Biocompatibility Evaluation In Vivo

The serum ALT, AST, BUN and Cre levels were analyzed according to the manufacturer's instructions for the assay kit. Besides, the major organ samples (heart, liver, spleen, lung, and kidney) were collected from mice for H&E staining to evaluate the histopathological changes.

### Statistical Analysis

The statistical significance difference was analyzed by a one‐way ANOVA followed by Tukey post hoc tests. The statistical significance was indicated as *p < 0.05, ** *p* < 0.01 and ***p < 0.001, ****p < 0.0001. Data are shown as Data are shown as mean ± SEM. The sample size in each experiment was specified in the figure legends.

## Conflict of Interest

The authors declare no conflict of interest.

## Supporting information



Supporting Information

## Data Availability

The data that support the findings of this study are available from the corresponding author upon reasonable request.
