# Supplementary material for: Traditional Chinese Medicine Borneol‐Based Polymeric Micelles Intracerebral Drug Delivery System for Precisely Pathogenesis‐Adaptive Treatment of Ischemic Stroke
Source: Adv Sci (Weinh). 2025 Jan 13;12(9):2410889. doi: 10.1002/advs.202410889 (PMC11884575; doi:10.1002/advs.202410889)
Supplement: Supplementary file 1 — Supporting Information [file ADVS-12-2410889-s001.pdf]

ADVANCED SCIENCE
Open Access

Supporting Information

for *Adv. Sci.*, DOI 10.1002/advs.202410889

Traditional Chinese Medicine Borneol-Based Polymeric Micelles Intracerebral Drug Delivery System for Precisely Pathogenesis-Adaptive Treatment of Ischemic Stroke

Yanan Wang, Xutao Ma, Xinyuan Wang, Liru Liu, Xue Zhang, Qiuyue Wang, Yingfei Zhu, Huanhua Xu, Liangmin Yu and Zhiyu He**

Supplementary Materials

Traditional Chinese medicine borneol-based polymeric micelles intracerebral drug delivery system for precisely pathogenesis-adaptive treatment of ischemic stroke

Yanan Wang, [1] *Xutao Ma,* [1] *Xinyuan Wang,* [1] *Liru Liu, Xue Zhang, Qiuyue Wang, Yingfei Zhu, Huanhua Xu* *, *Liangmin Yu, and Zhiyu He* *

Y.N. Wang, X.T. Ma, X.Y. Wang, L.R. Liu, X. Zhang, Q.Y. Wang, Y.F. Wang, L.M. Yu, Z.Y. He

Frontiers Science Center for Deep Ocean Multispheres and Earth Systems, Key Laboratory of Marine Chemistry Theory and Technology, Ministry of Education/Sanya Oceanographic Institution, Ocean University of China, Qingdao/Sanya 266003/572024, China.
Sanya Oceanographic Laboratory, Sanya 572024, China
College of Chemistry and Chemical Engineering, Ocean University of China, Qingdao, 266003, China.

H.H. Xu
Key Laboratory of Modern Preparation of TCM. Ministry of Education, Jiangxi University of Chinese Medicine, Nanchang 330004, China.
State Key Laboratory for the Modernization of Classical and Famous Prescriptions of Chinese Medicine. Jiangxi University of Chinese Medicine, Nanchang 330004, China.

[1] Contributed equally to this perspective paper.
E-mail: huanhua323@jxutcm.edu.cn (H.H. X*); hezhiyu@ouc.edu.cn (Z.Y. He*)

This file includes:

Supplementary Text

Materials and Methods
Figs. S1 to S2 Synthesis route of $p(PB)_{10}/(TB)_{30}$
Figs. S3 to S8 ^{1}H NMR spectra of MA-TK, MTB, MA-PEG, MPB, $p(PB)_{10}/(TB)_{30}$
Fig. S9 GPC spectra of $p(PB)_{10}/(TB)_{30}$
Figs. S10 to S12 Size distribution and stability of BA-loaded NPs
Fig. S13 Size distribution of Cy5-labeled NPs
Figs. S14 to S15 “Tyndall Effect” light path and TEM image of BA-loaded NPs after exposure to PBS medium containing 500 μM H_2O_2
Fig. S16 Cell toxicity of NPs *in vitro*
Figs. S17 to S20 Immunofluorescent images of ZO-1 and occludin
Fig. S21 to S22 Quantitative cell uptake results and cell uptake mechanism of NPs
Figs. S23 to S24 Flow cytometry results of intracellular ROS and Ca^{2+} levels
Figs. S25 to S26 Immunofluorescent images of Iba1, iNOS, and Arg-1
Fig. S27 The hemolysis rate evaluation of NPs
Fig. S28 The neurological assessment
Figs. S29 to S36 Immunofluorescent staining with NeuN, GFAP, Iba1, CD16/32, CD206, CD31, ZO-1, Occludin, and AQP4
Figs. S37 to S38 Biosafety evaluation
Tables S1 to S2 Reproducibility of different scaled batches and reproducibility of different batches using FNC platform

Materials and Methods

Synthesis of $p(BP)_{10}/(TB)_{30}$ polymer prodrug

Synthesis of TK

Briefly, 3-mercaptopropionic acid (5.3 g, 50 mmol, 1 eq) and anhydrous acetone (5.8 g, 100 mmol, 2 eq) were dissolved in a round-bottomed flask, followed by adding TFA (11.4 mg, 0.1 mmol, 0.002 eq) slowly with continuous stirring and then reacted for 8 hours at 25 °C. Then the reaction mixture was washed three times with cold n-hexane and cold deionized water and recrystallized to obtain white crystals. (5.8 g, 92% yield). ^{1}H NMR (600 MHz, Chloroform-*d*) δ 2.91 (t, *J* = 7.5 Hz, 2H), 2.68 (t, *J* = 7.4 Hz, 2H), 1.60 (s, 3H).

Synthesis of MA-TK

TK (5.4 g, 21.5 mmol, 1 eq) and 2-hydroxyethyl methacrylate (2.4 mL, 19.35 mmol, 0.9 eq) were dissolved in CH_2Cl_2 (40 mL) and then added sequentially with EDC·HCL (4.12 g, 21.5 mmol, 1 eq) and DMAP (788 mg, 6.45 mmol, 0.3 eq). After 12 hours of reaction at 25 °C, the reaction mixture was then added to saturated deionized water, which separated the aqueous phase with CH_2Cl_2. Subsequently, the organic phase was dried, filtered, concentrated, and then purified by silica gel column chromatography using ethyl acetate and petroleum ether (v/v, 1:6) as eluents to obtain a colorless and transparent liquid product (5,5,15-trimethyl-9,14-dioxo-10,13-dioxa-4,6-dithiahexadec-15-enoic acid, MA-TK) (6.7 g, yield 85%). ^{1}H NMR (600 MHz, Chloroform-*d*) δ 6.15 (t, *J* = 1.3 Hz, 1H), 5.61 (p, *J* = 1.7 Hz, 1H), 4.37 (s, 4H), 2.88 (t, *J* = 7.4 Hz, 2H), 2.87 (t, *J* = 7.4 Hz, 2H), 2.66 (q, *J* = 7.1 Hz, 4H), 1.95 (t, *J* = 1.3 Hz, 3H), 1.60 (s, 6H).

Synthesis of ROS-responsive hydrophobic MA-TK-Borneol monomer

MA-TK (2 g, 5.5 mmol, 1 eq) and Borneol (1.0 g, 6.6 mmol, 1.2 eq) were dissolved in 10 mL of THF, and the solution was chilled to 0 °C in an ice bath before adding EDC·HCL (1.3 g, 6.6 mmol, 1.2 eq) and DMAP (335 mg, 2.7 mmol, 0.5 eq). After 12 hours of reaction, the reaction mixture was evaporated to remove solvents, followed by being re-dissolved in CH_2Cl_2. Subsequently, the organic layer was rinsed successively with hydrochloric acid (HCl) (1 M), saturated sodium bicarbonate solution, and saltwater. The organic component was dried, dehydrated, and purified utilizing silica gel column chromatography with ethyl acetate and petroleum ether (v/v, 1:9) as eluent, resulting in MA-TK-Borneol (MTB) as a liquid substance (1.7 g, yield 61%). ^{1}H NMR (600 MHz, Chloroform-*d*) δ 6.15 (t, *J* = 1.3 Hz, 1H), 5.61 (p, *J* = 1.6 Hz, 1H), 4.91 (ddd, *J* = 10.0, 3.5, 2.2 Hz, 1H), 4.36 (s, 4H), 2.88 (t, *J* = 7.4 Hz, 2H), 2.87 (t, *J* = 7.4 Hz, 2H), 2.66 (t, *J* = 7.4 Hz, 2H), 2.63 (t, *J* = 7.3 Hz, 2H), 2.36 (tq, *J* = 10.5, 4.0 Hz, 1H), 1.95 (t, *J* = 1.3 Hz, 3H), 1.92 (ddd, *J* = 9.1, 4.6, 4.6 Hz, 1H), 1.74 (tdd, *J* = 16.0, 8.4, 4.0 Hz, 2H), 1.68 (dt, *J* = 9.3, 4.7 Hz, 1H), 1.60 (s, 6H), 1.30 (tdd, *J* = 12.2, 4.6, 2.2 Hz, 1H), 1.24 (ddd, *J* = 12.1, 9.4, 4.5 Hz, 1H), 0.90 (s, 3H), 0.87 (s, 3H), 0.84 (s, 3H).

Synthesis of MA-PEG

PEG (1 g, 1 mmol, 1 eq), DMAP (12 mg, 0.1 mmol, 0.1 eq) and methacrylic anhydride (15.4 mg, 1 mmol, 1 eq) were mixed in tetrahydrofuran (THF) (5 mL) and cooled to 0 °C with stirring until a clear solution was obtained. After continued reaction at room temperature for 4 hours, the

resulting solvent was concentrated on a rotary evaporator, precipitated by ice-cold ethyl ether (35 mL), and dried under vacuum, yielding a white powder of PEG-methacrylic acid (MA-PEG) (0.53 g, yield 49%).^{1}H NMR (600 MHz, Chloroform-*d*), δ 6.13 (s, 1H), 5.58 (q, 1H), 3.65 (d, 90H), 2.01 (t, 1H).

Synthesis of hydrophilic MA-PEG-borneol monomer

The CDI (162.2 mg, 1 mmol, 1 eq) was slowly added into a mixture of borneol (154.3 mg, 1 mmol, 1 eq) and MA-PEG$_{1k}$ (1.0 g, 1 mmol, 1 eq) in THF solution under a cold ice water bath. The reaction mixture was stirred at room temperature for 4 hours, followed by concentration by rotary evaporator, precipitation of ice-cold diethylether (35 mL), and drying under vacuum to obtain a white powder product of methacrylic acid-PEG-borneol (MA-PEG-Borneol, MPB) (0.38 g, 31% yield). ^{1}H NMR (600 MHz, Chloroform-*d*) δ 6.13 (s, 1H), 5.58 (q, 1H), 3.64 (s, 89H), 1.95 (t, 3H), 0.98 – 0.94 (m, 9H).

Synthesis and characterization of polymer prodrug

Synthesis of Cy5 labeled copolymer

Cy5 dye was conjugated to the polymer side chain via amide condensation. Briefly, p(BP)$_{10}$/(TB)$_{30}$ (40 mg), Cy5 amine (4.86 mg), and HOBT (0.1 mg) were combined in a DMSO solution (10 mL), followed by the slow dropwise addition of EDC·HCL (0.28 mg) in a DMSO solution. The resulting mixture was dialyzed against DMSO solution and water for 2 days each, and the final Cy5 labelled p(BP)$_{10}$/(TB)$_{30}$ (p(BP)$_{10}$/(TB)$_{30}$-Cy5) was obtained through freeze-drying. Similarly, p(P)$_{10}$/(TB)$_{30}$-Cy5 was synthesized by conjugating Cy5 dye to p(P)$_{10}$/(TB)$_{30}$ using the same procedure. mPEG$_{2K}$-b-PLGA$_{75K}$ (2 mg·mL^{-1}) was dissolved in THF and then added with 160 μL of CDI and 122 μL of a THF solution containing 0.24 mg of DMAP to activate the reaction substrate. The activated solution was then mixed with 326 μL of a solution containing 1.3 mg of Cy5 amine and reacted under dark conditions for 18 hours. The PEG-PLGA-Cy5 product was obtained after dialysis against a 50/50 (v/v) acetone/methanol solution and purification in a vacuum desiccator.

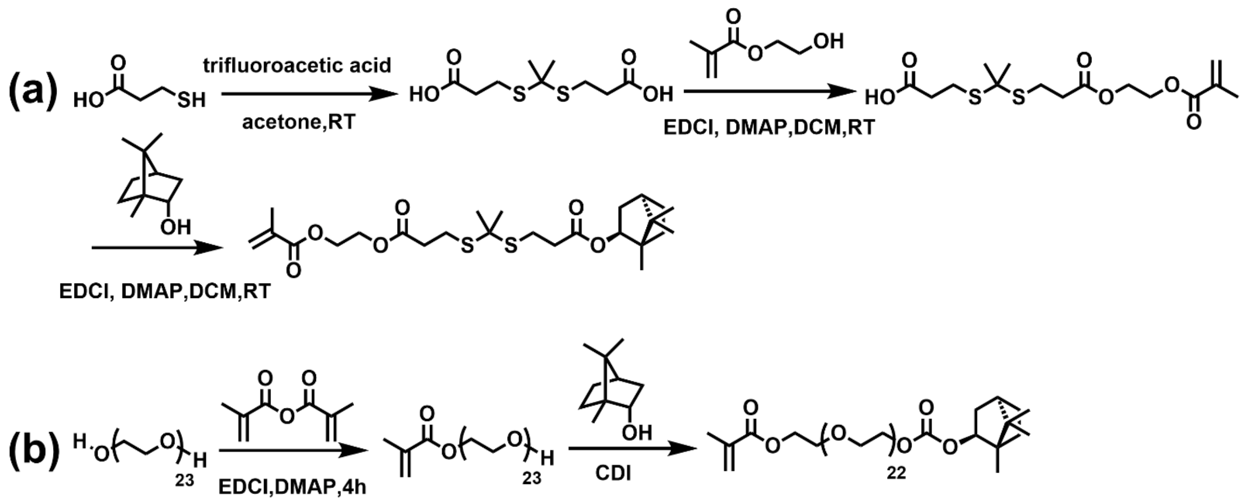

Fig. S1. Synthesis route of (a) ROS-cleavable hydrophobic methacrylate-TK-Borneol monomers (MTB) and (b) hydrophilic methacrylate-PEG-Borneol (MPB) monomers.

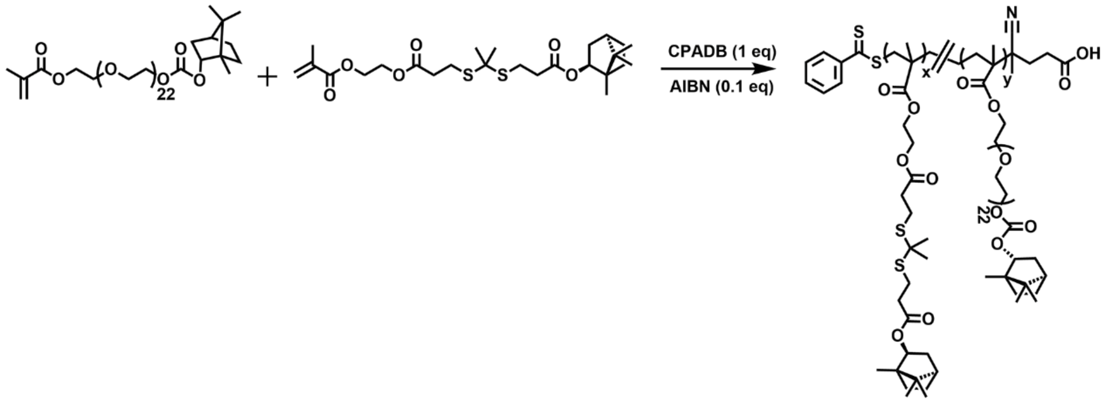

Fig. S2. Synthesis route of random copolymer $p(PB)_{10}/(TB)_{30}$.

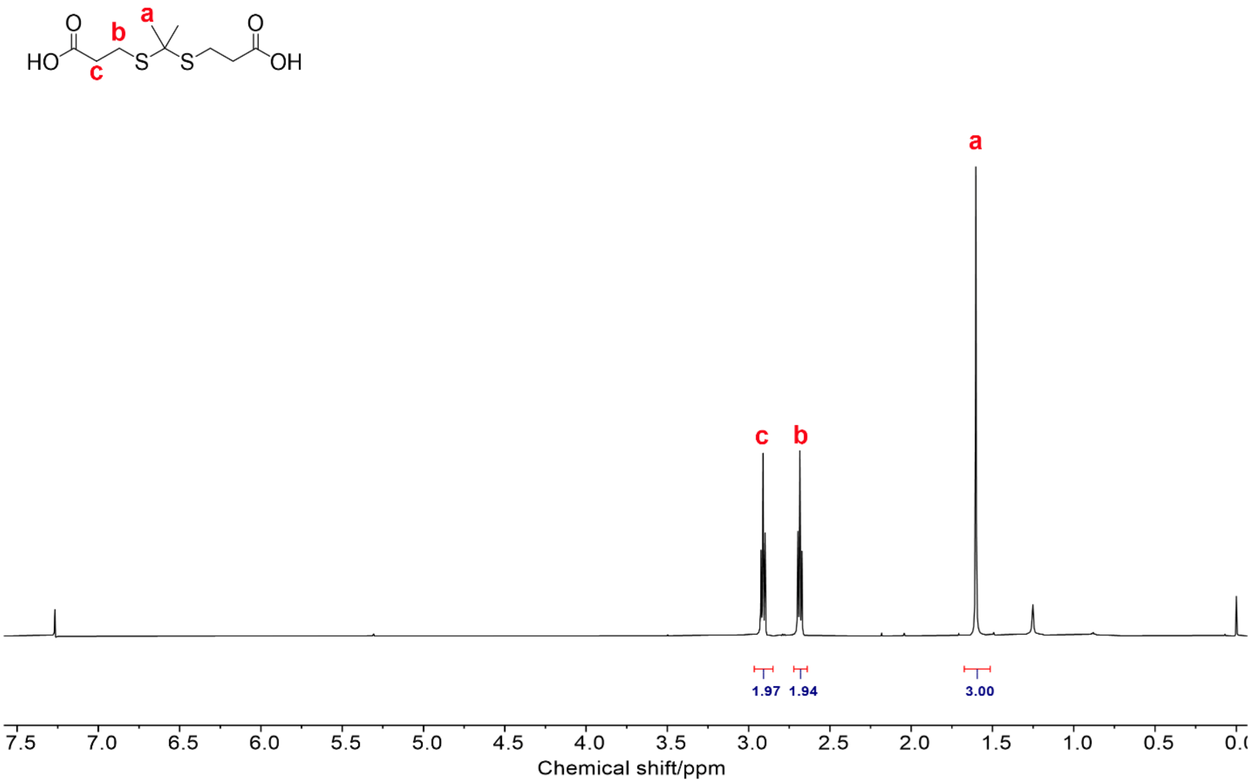

Fig. S3. ^{1}H NMR spectra of ROS-cleavable thioketal (TK) in $CDCl_3$.

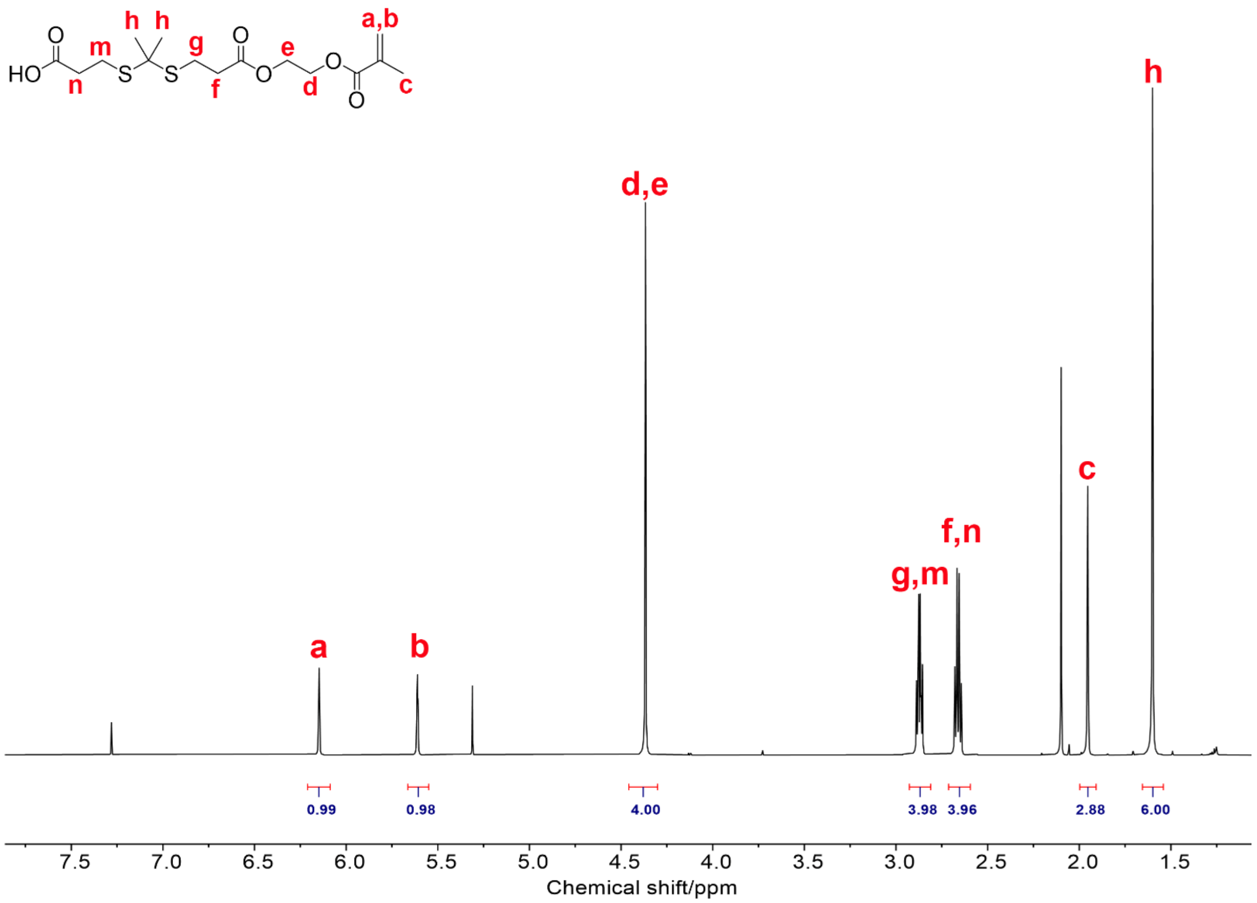

Fig. S4. ^{1}H NMR spectra of methacrylate-TK (MA-TK) in $CDCl_3$.

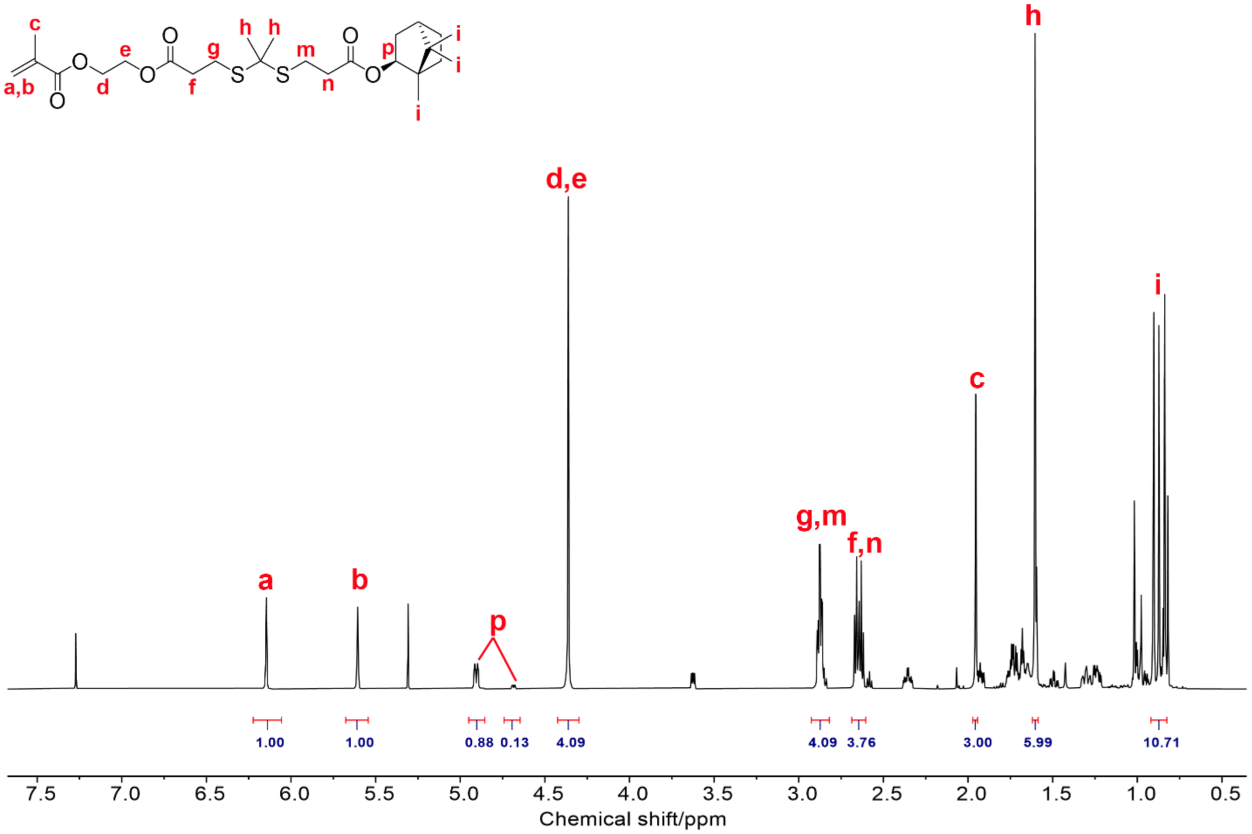

Fig. S5. ^{1}H NMR spectra of methacrylate-TK-Borneol monomers (MTB) in $CDCl_3$.

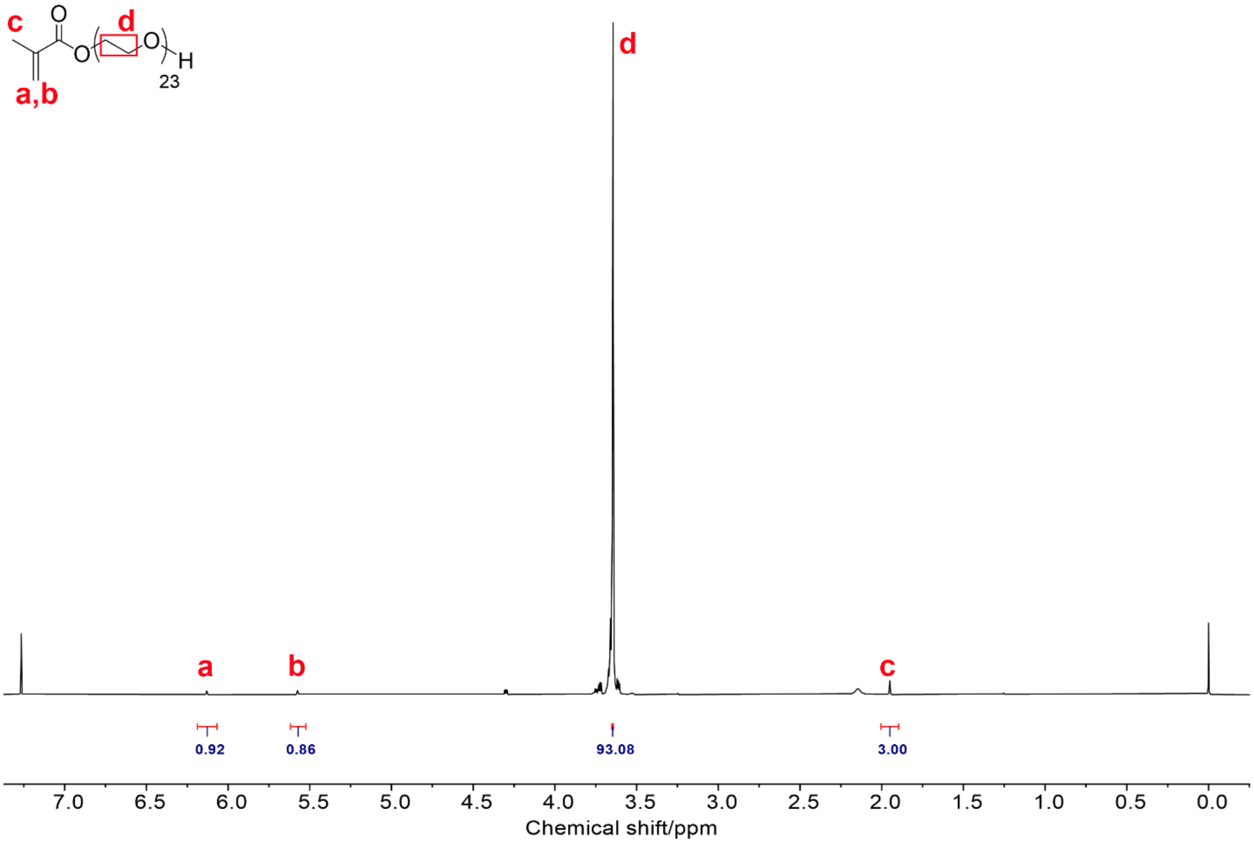

Fig. S6. ^{1}H NMR spectra of methacrylate-PEG (MP) in $CDCl_3$.

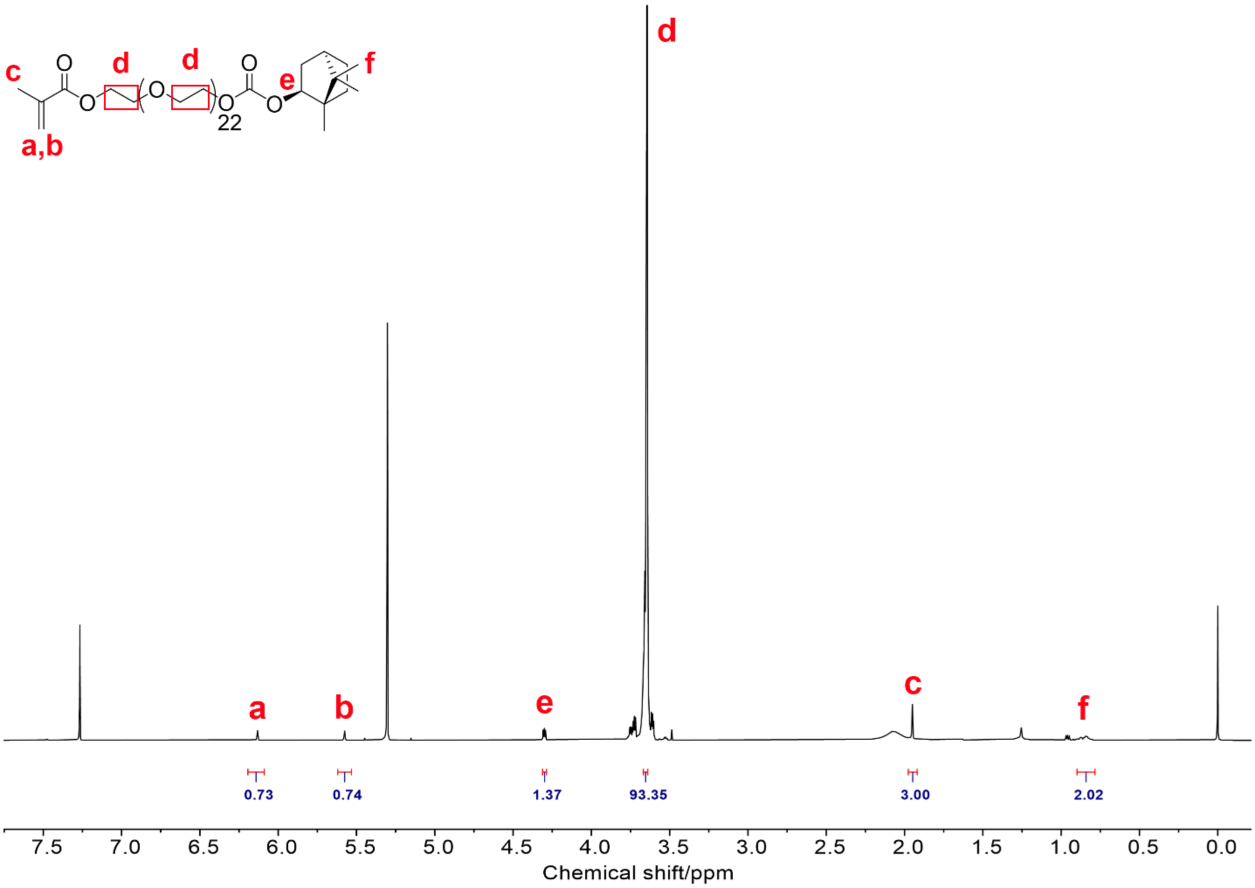

Fig. S7. ^{1}H NMR spectra of methacrylate-PEG-Borneol (MPB) in $CDCl_3$.

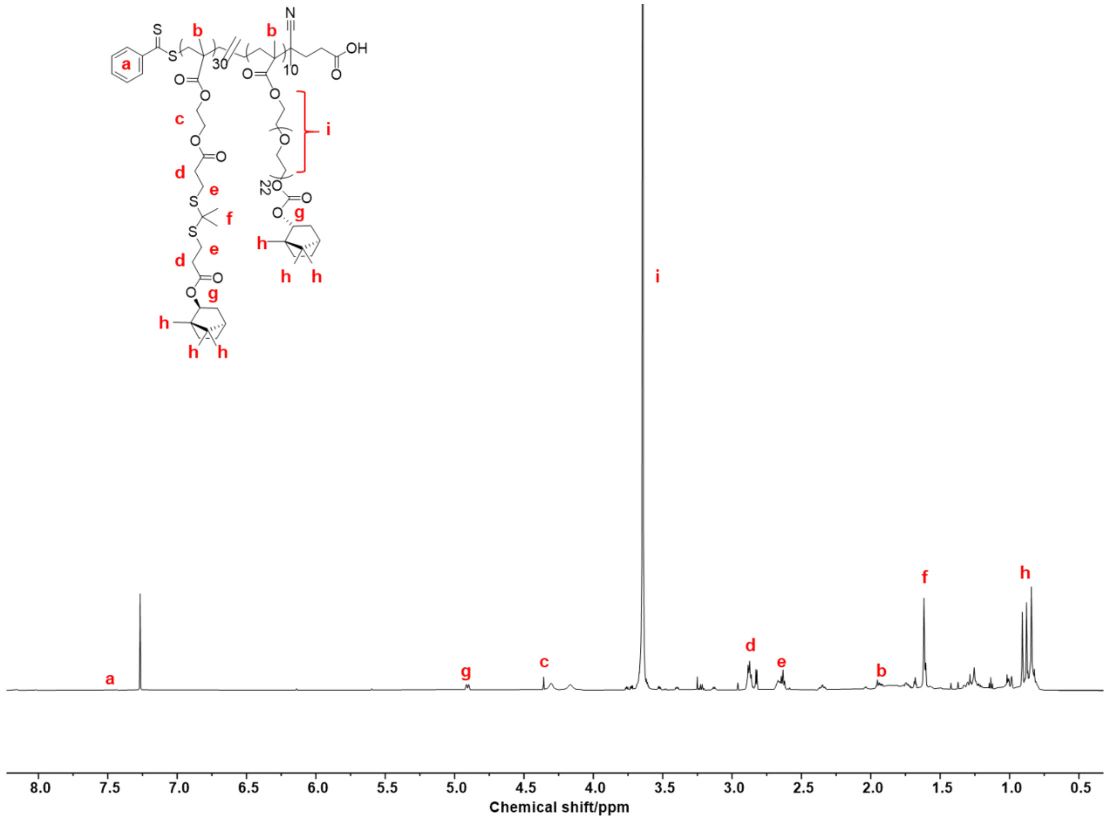

Fig. S8. The ^{1}H NMR spectra of p(PB)$_{10}$/(TB)$_{30}$ random copolymer in $CDCl_3$.

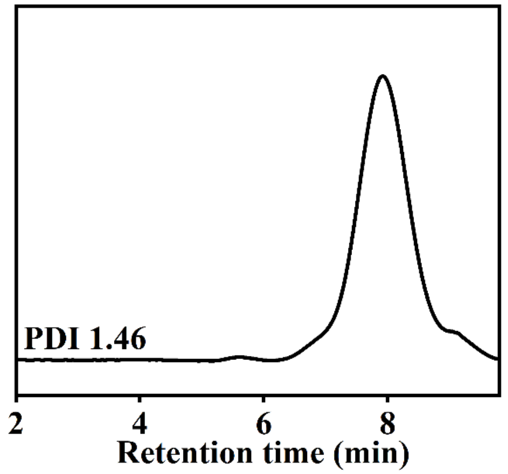

Fig. S9. GPC spectra of p(PB)$_{10}$/(TB)$_{30}$ random copolymer.

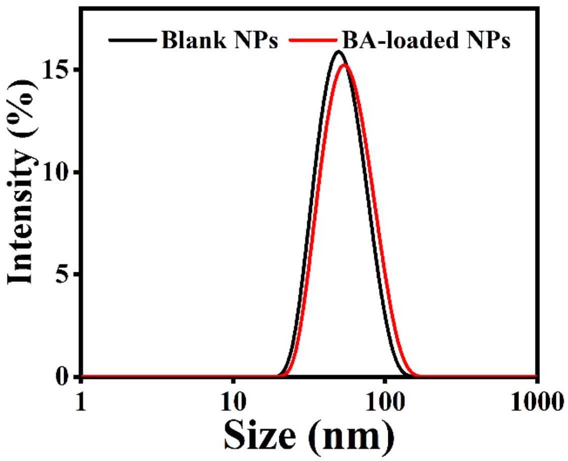

Fig. S10. Size distribution of blank $p(PB)_{10}/(TB)_{30}$ NPs and BA-loaded $p(PB)_{10}/(TB)_{30}$ NPs measured by DLS.

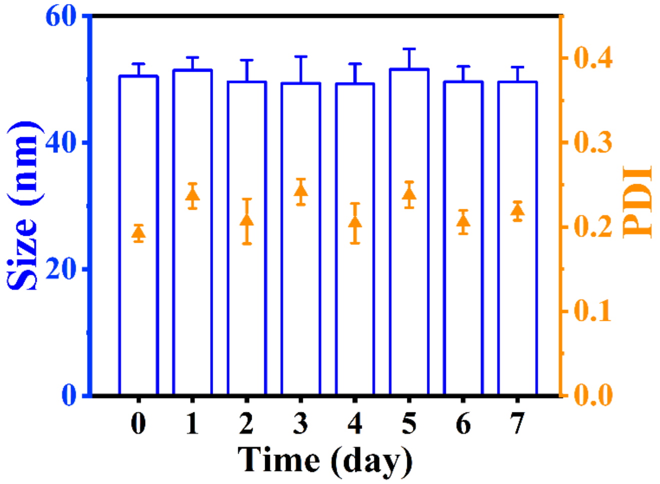

Fig. S11. Storage stability of BA-loaded NPs in water at 25 °C. Data are presented as means ± SEM, n = 3.

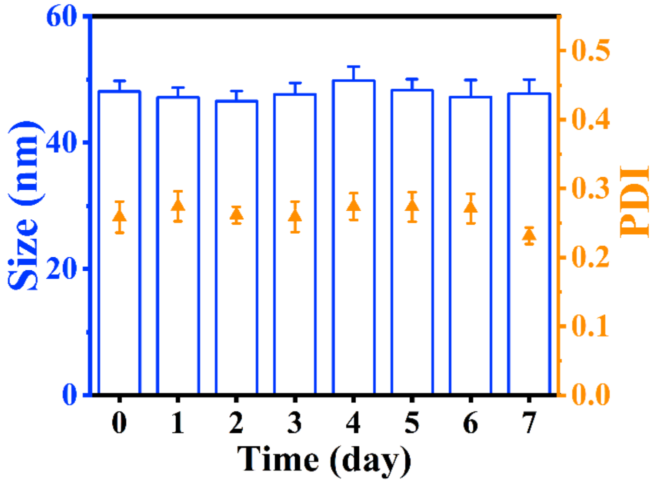

Fig. S12. The stability of blank NPs after 7-days incubation in PBS (10 mM, pH 7.4) medium, Data are presented as means ± SEM, n = 3.

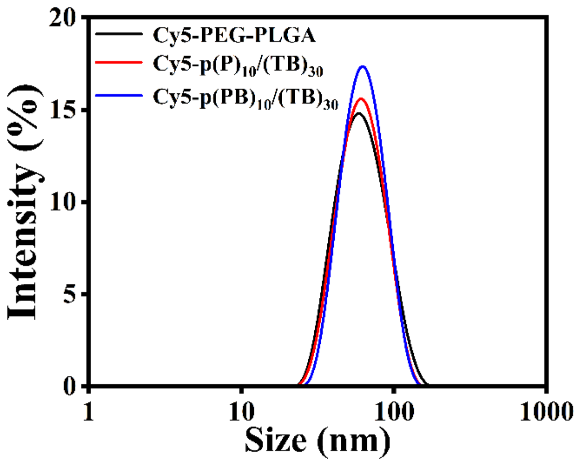

Fig. S13. The size distribution of Cy5-PEG-PLGA, Cy5-p(P)$_{10}$/(TB)$_{30}$ Cy5-p(PB)$_{10}$/(TB)$_{30}$. Data are presented as means ± SEM, n = 3.

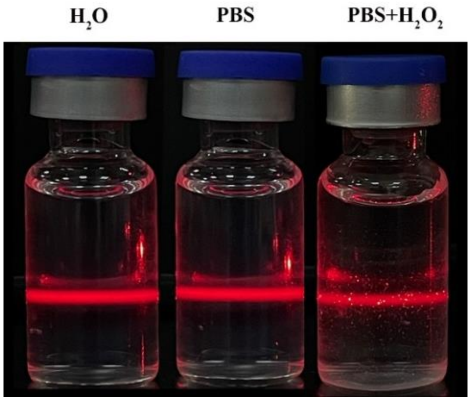

Fig. S14. The 'Tyndall Effect' light path of NPs after exposure to ddH_2O, PBS medium (10 mM, pH 7.4) and PBS medium (10 mM, pH 7.4) containing 500 μM H_2O_2.

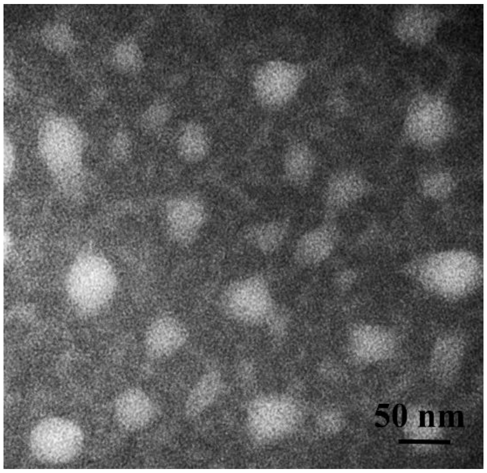

Fig. S15. The representative TEM image of NPs after exposure to PBS medium (10 mM, pH 7.4) containing 500 μM H_2O_2 for 60 min. n = 3.

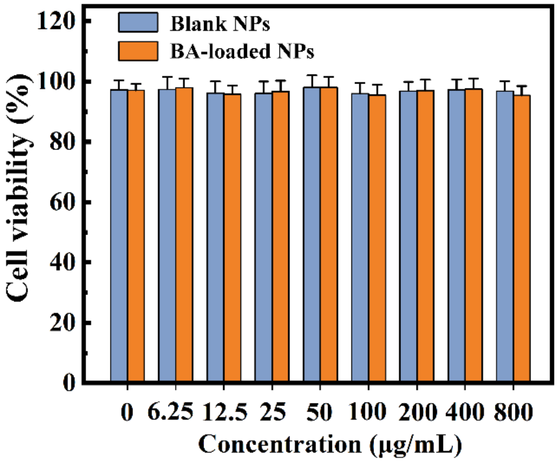

Fig. S16. The cell viability of SH-SY5Y cells after incubated with blank NPs and BA-loaded NPs at polymer concentration from 0-800 μg·mL^{-1} for evaluating the cell toxicity of NPs Data are presented as means ± SEM, n = 3.

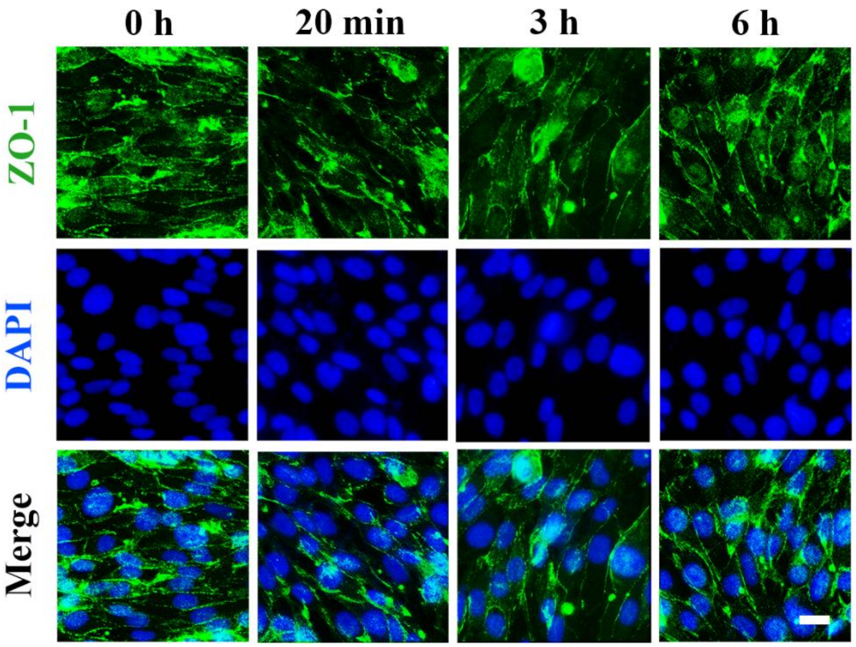

Fig. S17. Representative fluorescent image of the variations in ZO-1 proteins over time in BBB-mimic bEnd.3 monolayer following incubation with $p(P)_{10}/(TB)_{30}$ NPs. Scale bar: 20 μm.

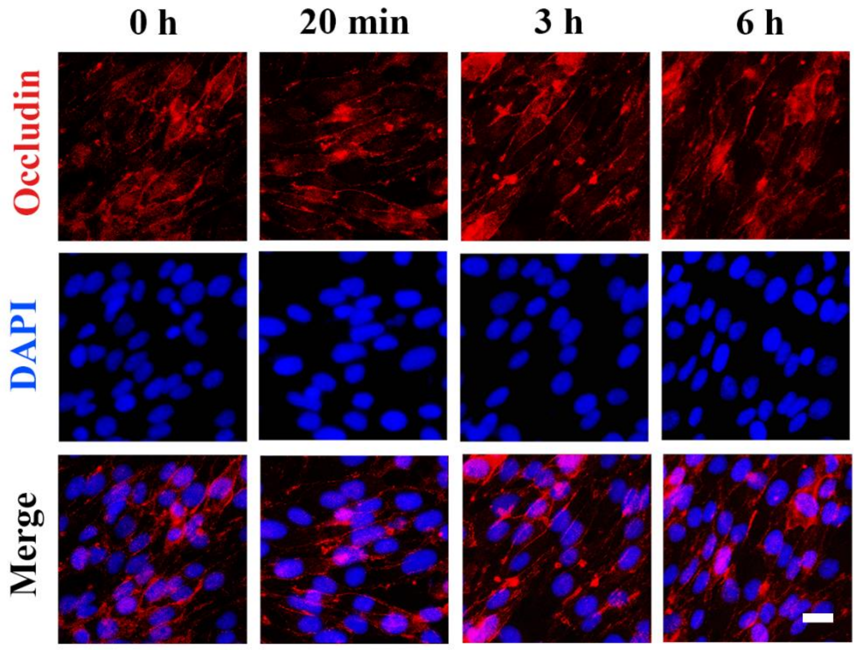

Fig. S18. Representative fluorescent image of the variations in Occludin proteins over time in BBB-mimic bEnd.3 monolayer following incubation with $p(P)_{10}/(TB)_{30}$ NPs. Scale bar: 20 μm.

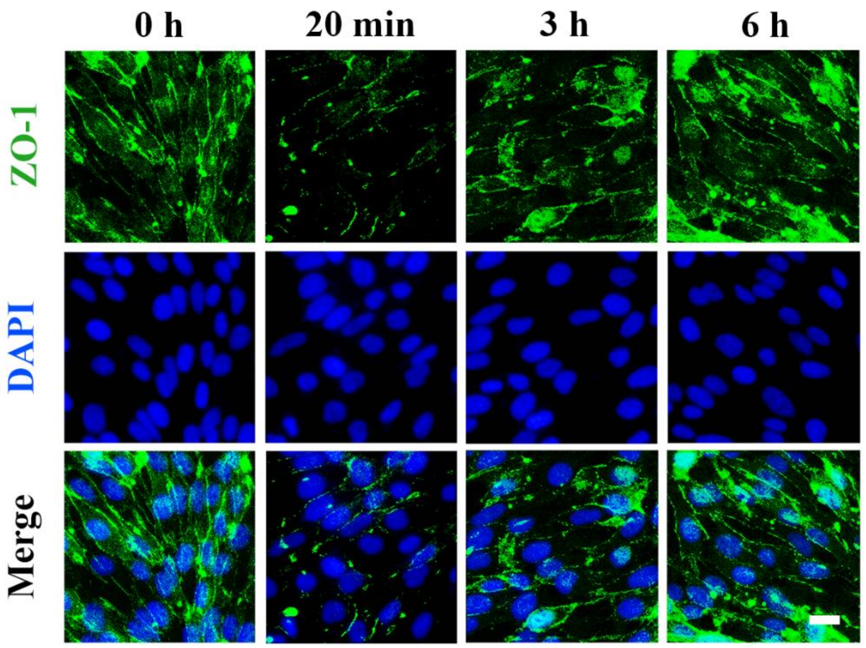

Fig. S19. Representative fluorescent image of the variations in ZO-1 proteins over time in BBB-mimic bEnd.3 monolayer following incubation with p(PB)$_{10}$/(TB)$_{30}$ NPs. Scale bar: 20 μm.

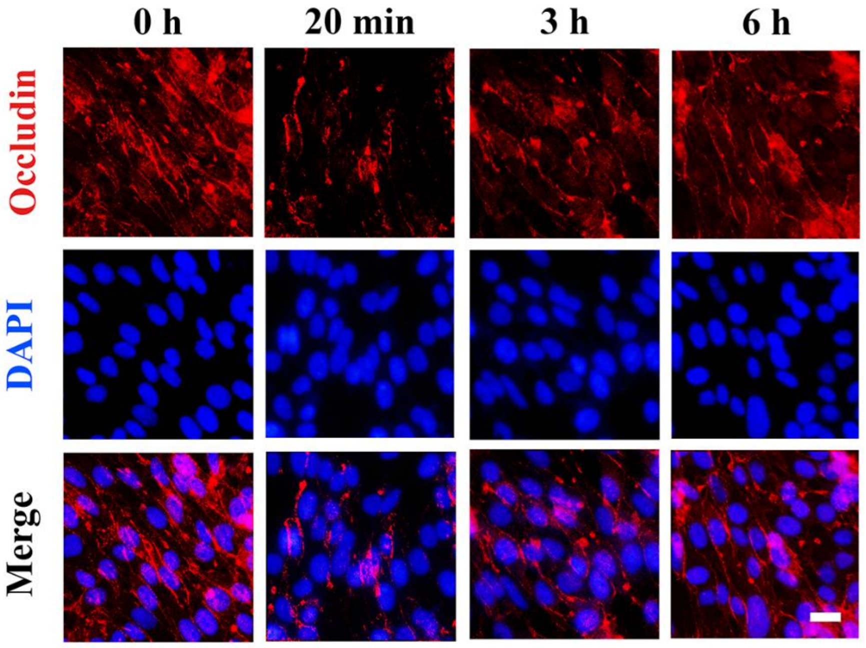

Fig. S20. Representative fluorescent image of the variations in occludin proteins over time in BBB-mimic bEnd.3 monolayer following incubation with p(PB)$_{10}$/(TB)$_{30}$ NPs. Scale bar: 20 μm.

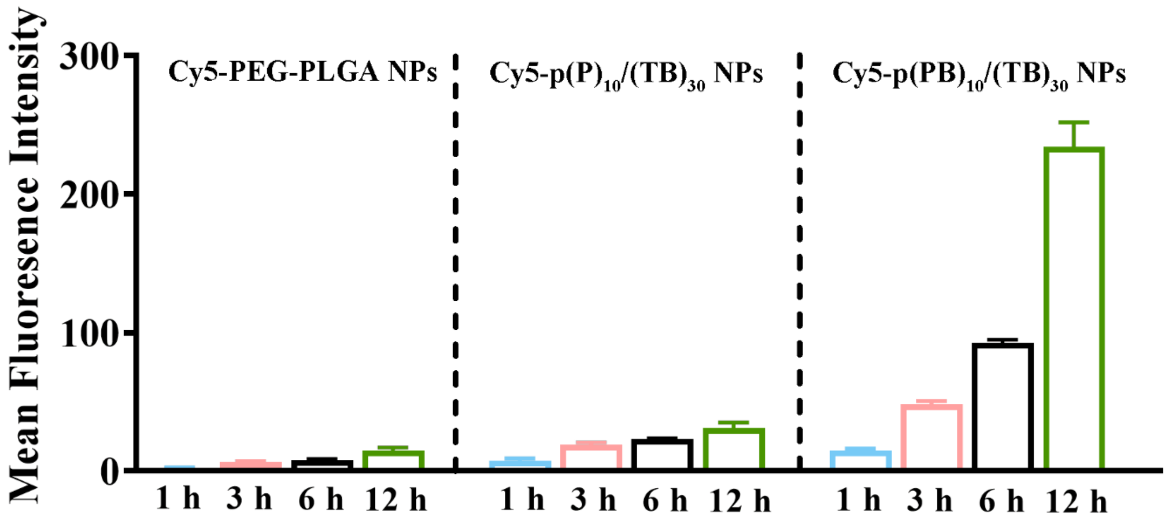

Fig. S21. The semi-quantitative cell uptake results of Cy5-labelled NPs in SH-SY5Y cells after crossing the BBB-mimic bEnd.3 monolayer were assessed using confocal microscopy imaging. Data are presented as means ± SEM, n = 6.

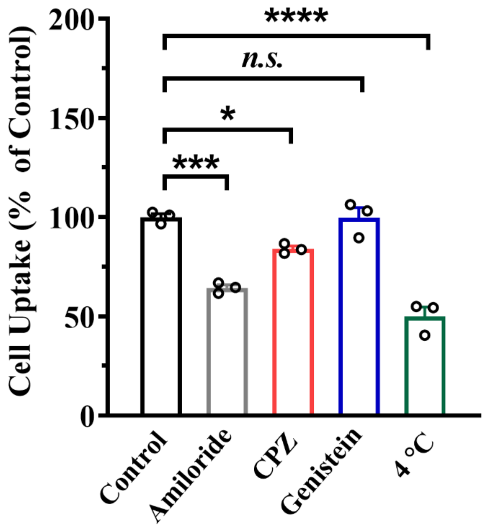

Fig. S22. the percentage of cell uptake of Cy5-labeled Cy5-p(PB)$_{10}$/(TB)$_{30}$ NPs in SH-SY5Y cells following treatment of uptake inhibitor. n = 3. CPZ, amiloride, and genistein referred to clathrin-mediated endocytosis inhibitor (chlorpromazine), macropinocytosis-dependent endocytosis inhibitor and caveolae-mediated endocytosis inhibitor, respectively.

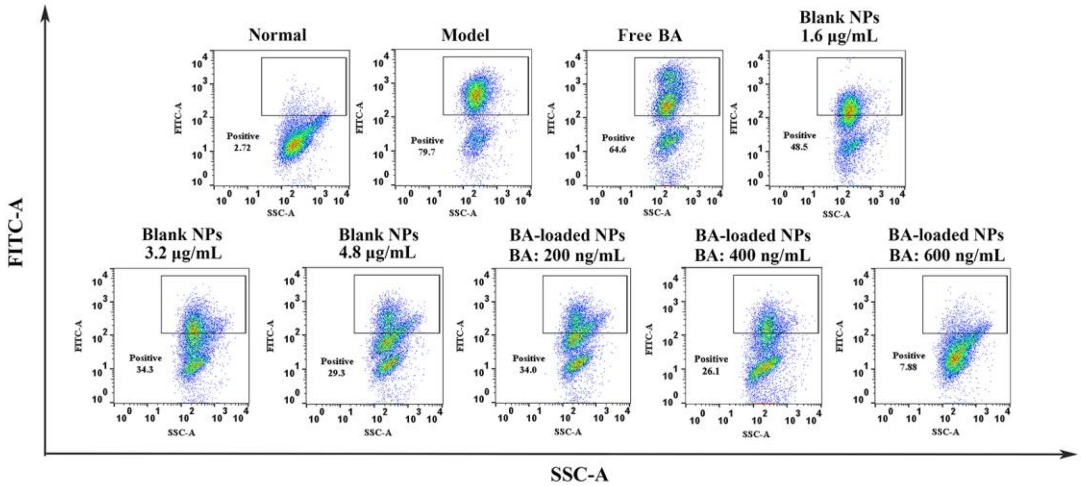

Fig. S23. Representative flow cytometry results for analyzing intracellular ROS levels in the glutamate-induced excitotoxicity acute neuronal cell injury model (SH-SY5Y cells) after treatment with different formulations. n = 3.

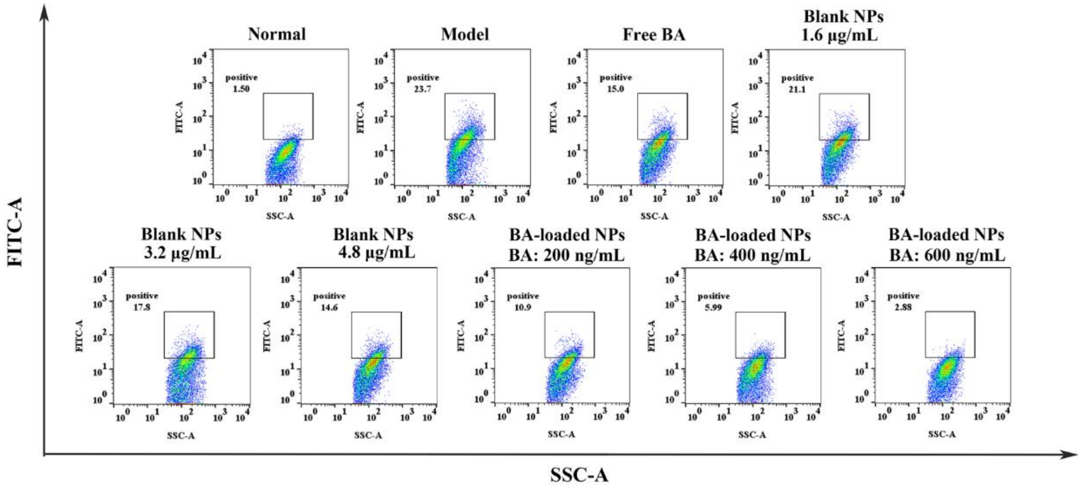

Fig. S24. Representative flow cytometry results for analyzing intracellular Ca^{2+} levels in the glutamate-induced excitotoxicity acute neuronal cell injury model (SH-SY5Y cells) after treatment with different formulations. n = 3.

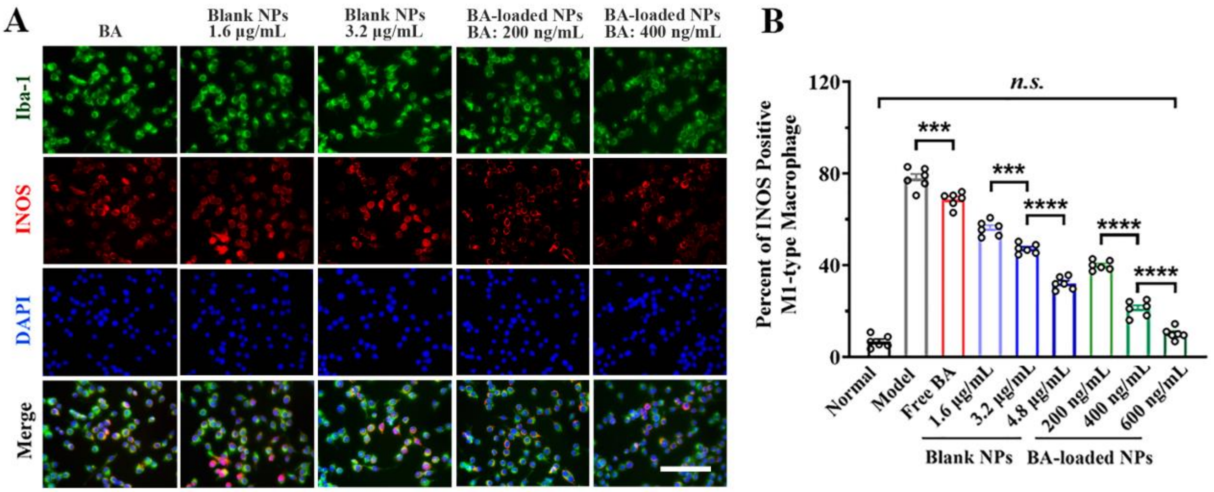

Fig. S25. (A) Representative immunofluorescent results of pan-microglia marker (Iba1) and pro-inflammatory M1-microglia marker (iNOS) for evaluating the M1 phenotype polarization of microglia (BV2 cells). Scale bar: 100 μm. (B) The semi-quantitative MFI results of iNOS for analyzing the number of M1 phenotype microglia. Data are presented as means ± SEM, n = 6.

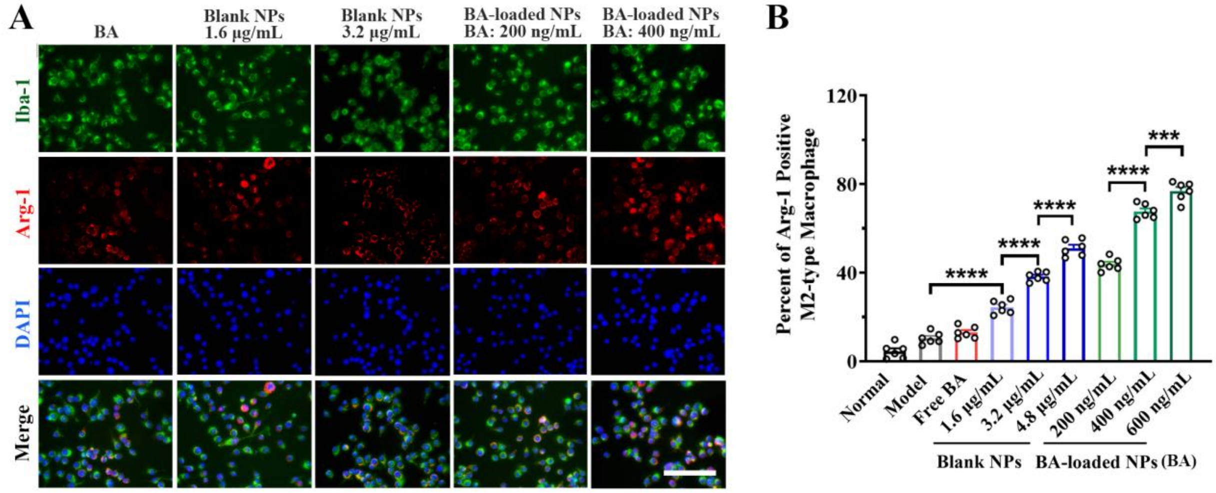

Fig. S26. (A) Representative immunofluorescent results of pan-microglia marker (Iba1) and anti-inflammatory M2-microglia marker (Arg-1) for evaluating the M2 phenotype polarization of microglia (BV2 cells). Scale bar: 100 μm. (B) The semi-quantitative MFI results of iNOS for analyzing the number of M1 phenotype microglia. Data are presented as means ± SEM, n = 6.

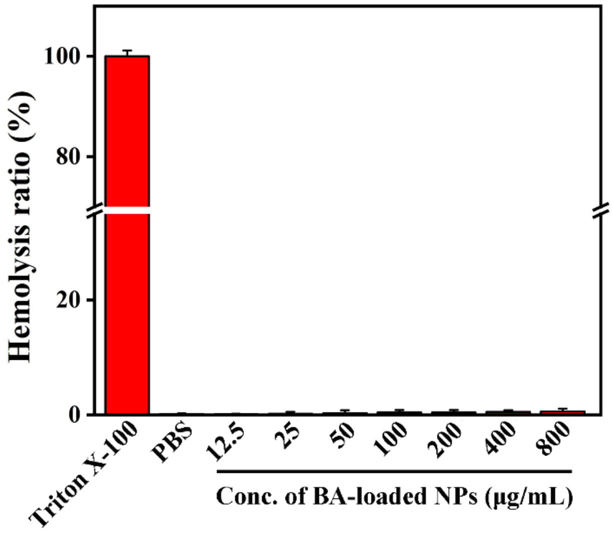

Fig. S27. The hemolysis rate of red blood cells incubated with different polymer prodrug concentrations of BA-loaded NPs. The polymer dose ranged from 12.5 to 800 μg·mL^{-1}. Data are presented as means ± SEM, n = 3.

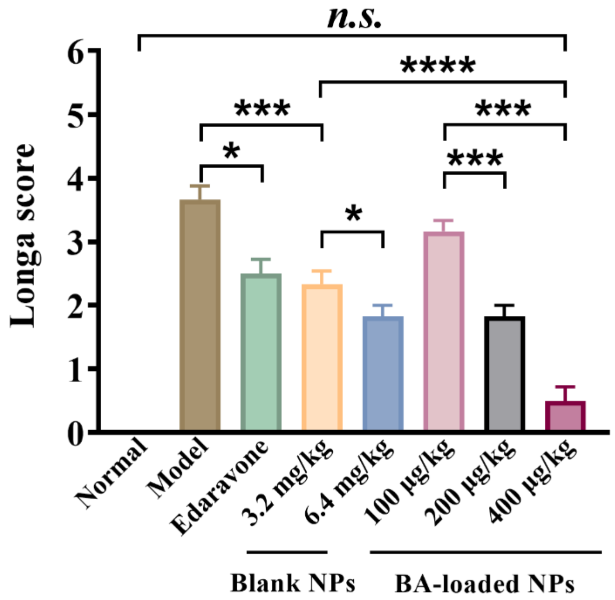

Fig. S28. The neurological assessment of MCAO mice treated with Blank NPs (polymer prodrug dose: 6.4 mg·kg^{-1}) and BA-loaded NPs (BA dose: 100, 200, and 400 μg·kg^{-1}, corresponding polymer dose: 0.8, 1.6, and 3.2 mg·kg^{-1}) according to Longa's five-point scale. Data are presented as means ± SEM, n = 3.

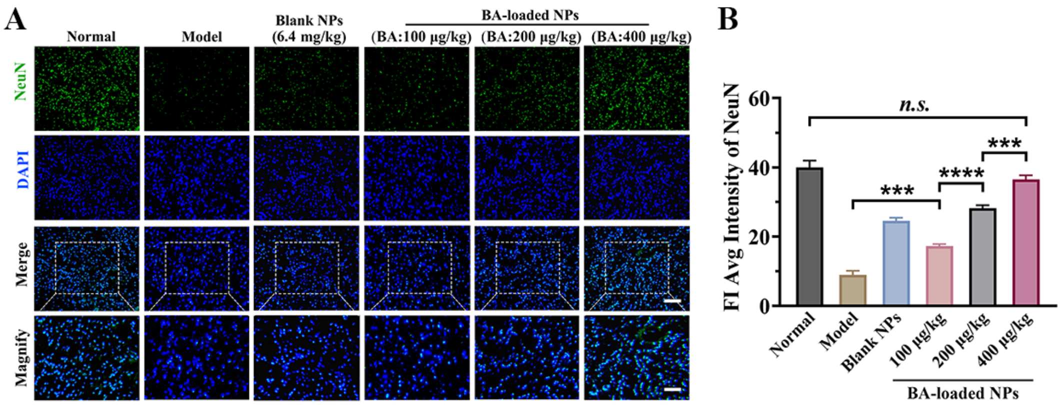

Fig. S29. (A) Immunofluorescence staining with NeuN, a marker for neurons, in infarct areas of MCAO mice treated with Blank NPs (polymer prodrug dose: 6.4 mg·kg^{-1}) and BA-loaded NPs (BA dose: 100, 200, and 400 μg·kg^{-1}, corresponding polymer dose: 0.8, 1.6, and 3.2 mg·kg^{-1}) and (B) the semi-quantitative fluorescent intensity results of NeuN. Scale bar: merge (100 μm), magnify (50 μm). Data are presented as means ± SEM, n = 6.

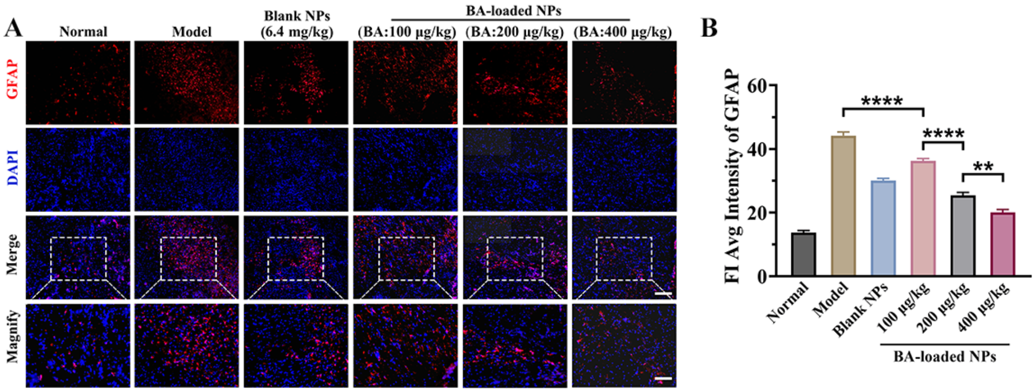

Fig. S30. (A) Representative immunofluorescence staining with GFAP, a marker for activated astrocytes, in infarct areas of MCAO mice treated with Blank NPs (polymer prodrug dose: 6.4 mg·kg^{-1}) and BA-loaded NPs (BA dose: 100, 200, and 400 μg·kg^{-1}, corresponding polymer dose: 0.8, 1.6, and 3.2 mg·kg^{-1}) and (B) the semi-quantitative fluorescent intensity results of GFAP. Scale bar: merge (100 μm), magnify (50 μm). Data are presented as means ± SEM, n = 6.

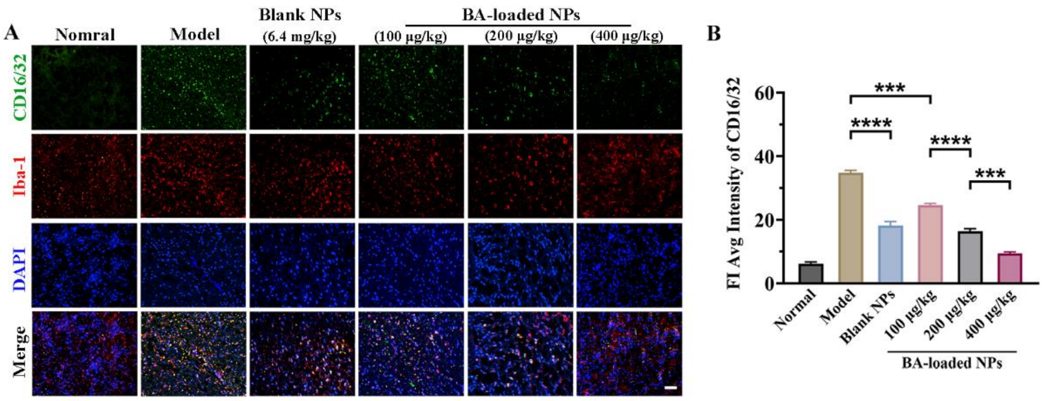

Fig. S31. (A) Representative immunofluorescent results of pan-microglia marker (Iba1) and pro-inflammatory M1-microglia marker (CD16/32) for evaluating the M1 phenotype polarization of microglia in MCAO mice Blank NPs (polymer prodrug dose: 6.4 mg·kg^{-1}) and BA-loaded NPs (BA dose: 100, 200, and 400 μg·kg^{-1}, corresponding polymer dose: 0.8, 1.6, and 3.2 mg·kg^{-1}). Scale bar: 50 μm. (B) the semi-quantitative fluorescent intensity results of CD16/32. Data are presented as means ± SEM, n = 6.

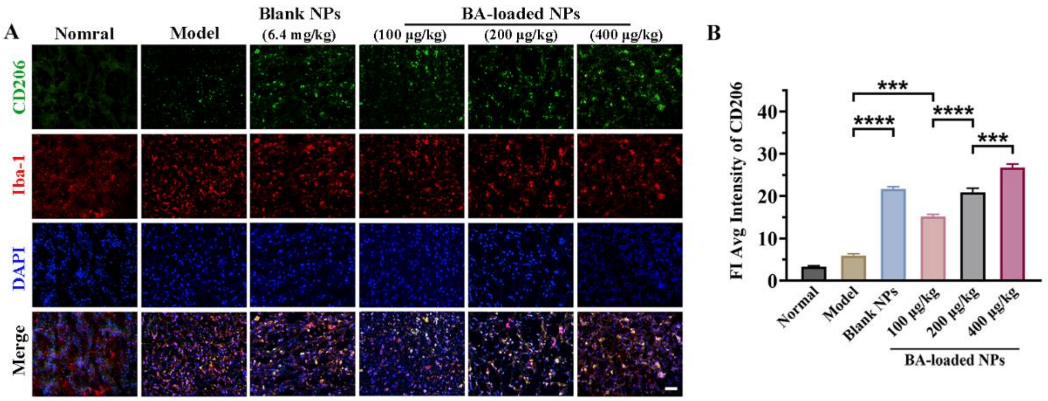

Fig. S32. Representative immunofluorescent results of pan-microglia marker (Iba1) and pro-inflammatory M2-microglia marker (CD206) for evaluating the M2 phenotype polarization of microglia in MCAO mice Blank NPs (polymer prodrug dose: 6.4 mg·kg^{-1}) and BA-loaded NPs (BA dose: 100, 200, and 400 μg·kg^{-1}, corresponding polymer dose: 0.8, 1.6, and 3.2 mg·kg^{-1}). Scale bar: 50 μm. (B) the semi-quantitative fluorescent intensity results of CD206. Data are presented as means ± SEM, n = 6.

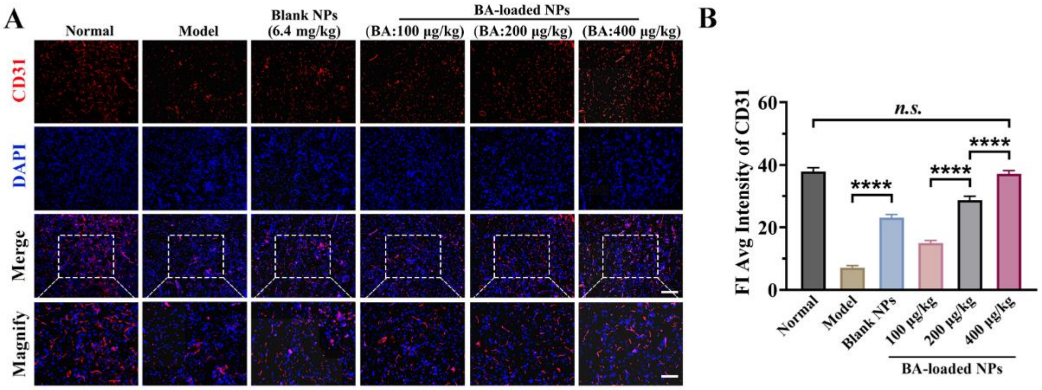

Fig. S33. (A) Representative immunofluorescent results of CD31, a biomarker for endothelial cells for evaluating recovery of microvessel endothelium in MCAO mice with Blank NPs (polymer prodrug dose: 6.4 mg·kg^{-1}) and BA-loaded NPs (BA dose: 100, 200, and 400 μg·kg^{-1}, corresponding polymer dose: 0.8, 1.6, and 3.2 mg·kg^{-1}). Scale bar: merge (200 μm), magnify (100 μm). (B) The semi-quantitative MFI results of CD31. Data are presented as means ± SEM, n = 6.

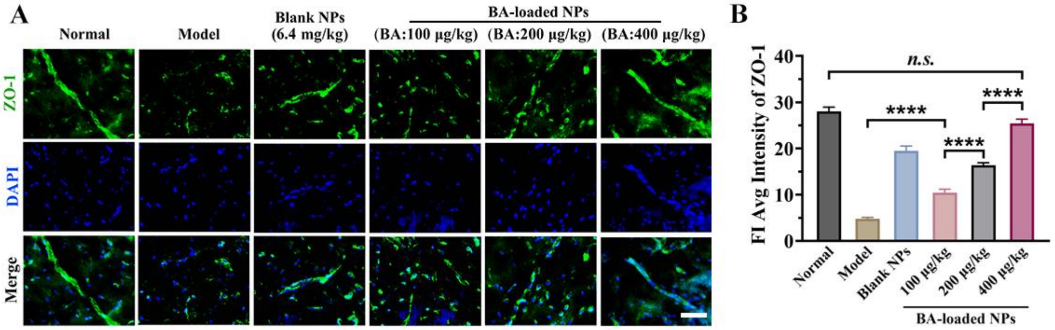

Fig. S34. (A) Representative immunofluorescent results of key TJs' protein (ZO-1) that maintain BBB integrity in MCAO mice treated with Blank NPs (polymer prodrug dose: 6.4 mg·kg^{-1}) and BA-loaded NPs (BA dose: 100, 200, and 400 μg·kg^{-1}, corresponding polymer dose: 0.8, 1.6, and 3.2 mg·kg^{-1}). Scale bar: 50 μm. (B) The semi-quantitative MFI results of ZO-1. Data are presented as means ± SEM, n = 6.

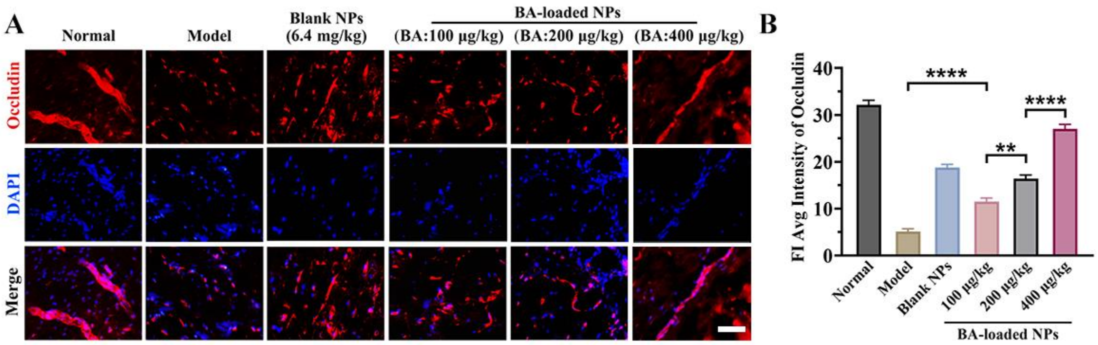

Fig. S35. (A) Representative immunofluorescent results of key TJs' protein (Occludin) that maintain BBB integrity in MCAO mice treated with Blank NPs (polymer prodrug dose: 6.4 mg·kg^{-1}) and BA-loaded NPs (BA dose: 100, 200, and 400 μg·kg^{-1}, corresponding polymer dose: 0.8, 1.6, and 3.2 mg·kg^{-1}). Scale bar: 50 μm (B) The semi-quantitative MFI results of Occudin. Data are presented as means ± SEM, n = 6.

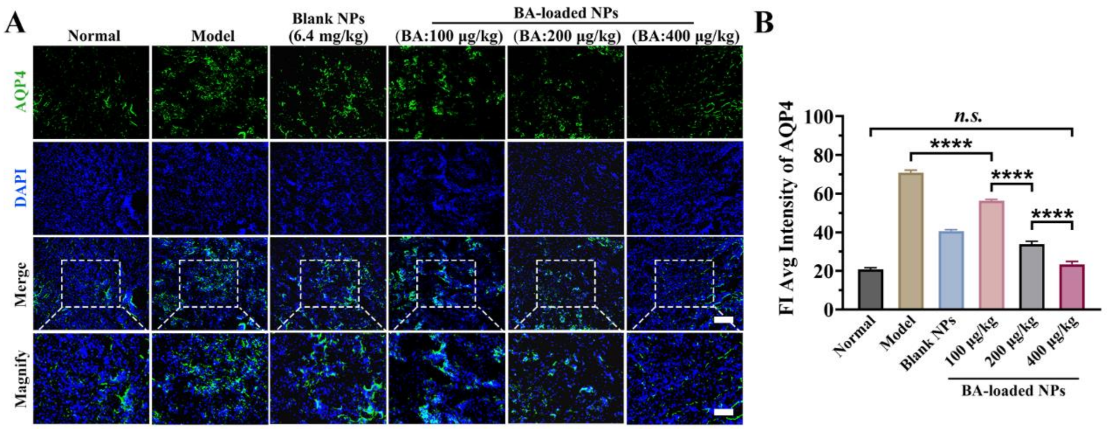

Fig. S36. (A) Representative immunofluorescent results of AQP4 in MCAO mice treated with Blank NPs (polymer prodrug dose: 6.4 mg·kg^{-1}) and BA-loaded NPs (BA dose: 100, 200, and 400 μg·kg^{-1}, corresponding polymer dose: 0.8, 1.6, and 3.2 mg·kg^{-1}). Scale bar: merge (200 μm), magnify (100 μm). (B) The semi-quantitative MFI results of AQP4. Data are presented as means ± SEM, n = 6.

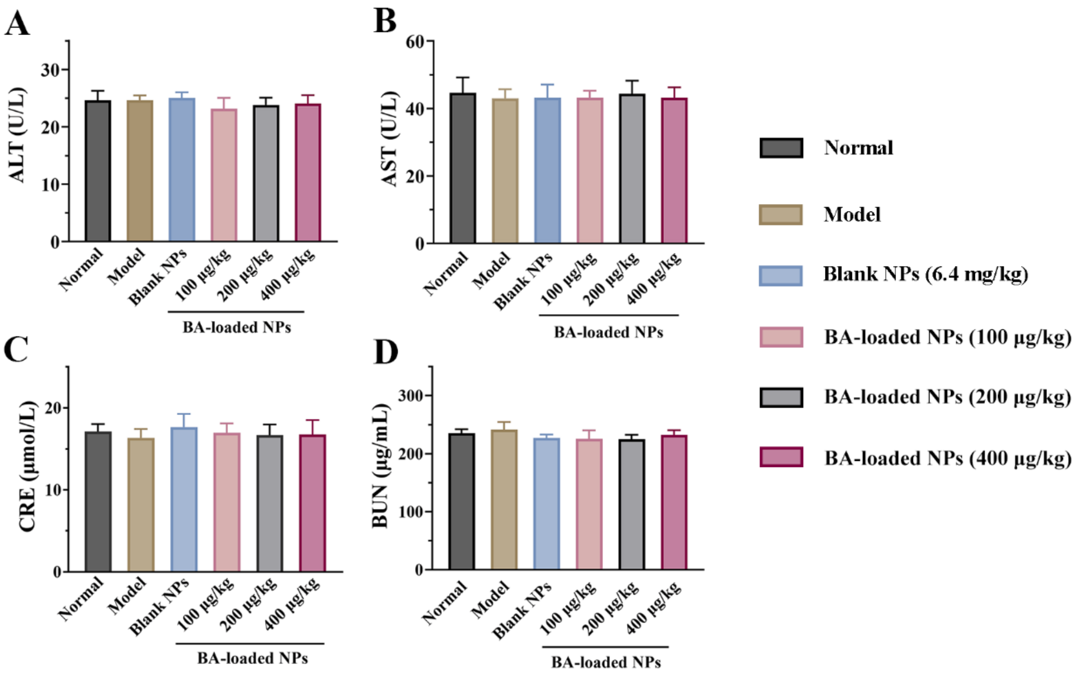

Fig. S37. The serum levels of (A) ALT, (B) AST, (C) CRE and (D) BUN. Data are presented as means ± SEM, n = 8-12

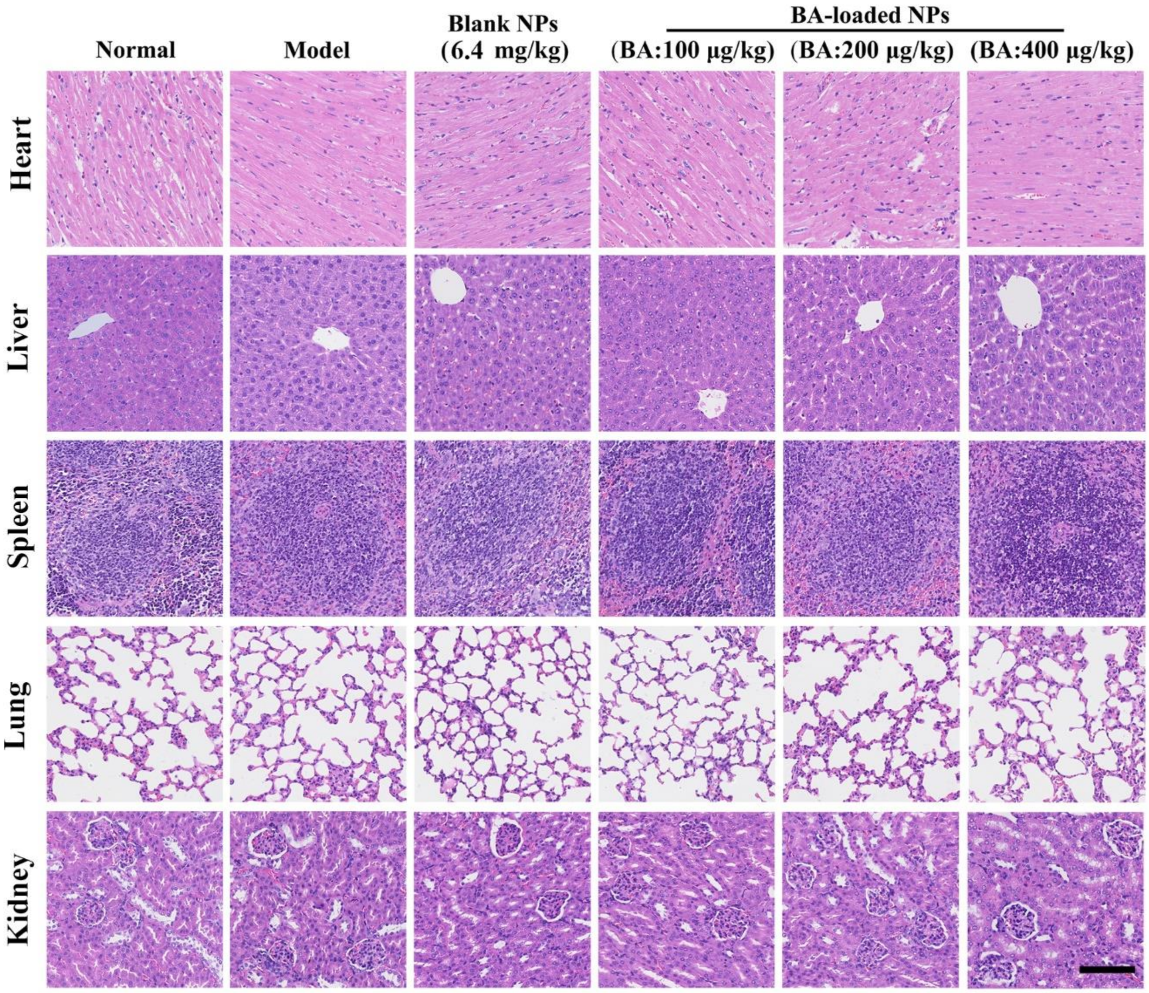

Fig. S38. The representative H&E staining of major organs from mice treated with different formulations. Scale bar: 100 μm.

Table S1. Reproducibility of different scaled batches (2 ~ 100 mL)

**Batch size (mL)**	**2**	**10**	**25**	**50**	**100**
**Average size (nm)**	53.6 ± 2.13	51.9 ± 1.57	50.9 ± 1.73	50.5 ± 2.84	50.3 ± 0.96
**PDI**	0.22 ± 0.02	0.16 ± 0.03	0.16 ± 0.03	0.14 ± 0.01	0.14 ± 0.01
**EE**	82.8 ± 1.6%	83.7 ± 1.6%	82.3 ± 0.7%	82.7 ± 2.2%	82.8 ± 2.6%

Table S2. Reproducibility of different batches (Sample #1 ~ Sample #5)

**Batch**	**1#**	**2#**	**3#**	**4#**	**5#**
**Average size (nm)**	53.4 ± 1.26	57.5 ± 3.39	56.9 ± 4.38	56.9 ± 0.17	55.8 ± 0.53
**PDI**	0.20 ± 0.03	0.22 ± 0.01	0.24 ± 0.02	0.27 ± 0.03	0.17 ± 0.05
**EE**	83.1 ± 3.9%	81.8 ± 1.2%	80.4 ± 3.5%	83.2 ± 0.5%	83.0 ± 3.5%
